# Mutant p53 stimulates cell invasion through an interaction with Rad21 in human ovarian cancer cells

**DOI:** 10.1038/s41598-017-08880-4

**Published:** 2017-08-22

**Authors:** Ji-Hye Ahn, Tae Jin Kim, Jae Ho Lee, Jung-Hye Choi

**Affiliations:** 1 Department of Life and Nanopharmaceutical Sciences, Kyung Hee University, Seoul, 02447 South Korea; 2 Division of Molecular Biology, College of Pharmacy, Kyung Hee University, Seoul, 02447 South Korea; 3 Department of Obstetrics and Gynecology, Cheil General Hospital and Women’s Healthcare Center, Dankook University College of Medicine, Seoul, 04619 South Korea; 4 Laboratory of Molecular Oncology, Cheil General Hospital and Women’s Healthcare Center, Dankook University College of Medicine, Seoul, 04619 South Korea

## Abstract

Missense mutations of TP53 are extremely common, and mutant p53 accumulation and gain-of-function play crucial roles in human ovarian cancer. Here, we investigated the role of mutant p53 in cell migration and invasion as well as its underlying molecular mechanisms in human ovarian cancer cells. Overexpression of mutant p53 significantly increased migration and invasion in p53-null SKOV3 cells. In contrast, knockdown of mutant p53 significantly compromised mutant p53-induced cell migration and invasion. Microarray analysis revealed that several migration/invasion-related genes, including S1PR1 (Sphingosine-1-phosphate receptor 1) and THBS1 (Thrombospodin 1), were significantly upregulated in SKOV3 cells that overexpressed mutant p53-R248 (SKOV3^R248^). We found that Rad21 is involved in the transcriptional regulation of the migration/invasion-related genes induced by mutant p53-R248. Knockdown of Rad21 significantly attenuated the mutant p53-R248-induced invasion and the expressions of S1PR1 and THBS1. Moreover, co-immunoprecipitation and chromatin immunoprecipitation assays revealed that mutant p53 interacts with Rad21 and binds to the Rad21-binding elements in the S1PR1 and THBS1 genes. Finally, downregulation of S1PR1 significantly attenuated the invasion driven by mutant p53-R248. These novel findings reveal that mutant p53-R248 maintains gain-of-function activity to stimulate cell invasion and induces the related gene expressions through an interaction with Rad21 in human ovarian cancer cells.

## Introduction

Ovarian cancer is one of the most common malignancies and is the fifth leading cause of cancer-related deaths among women in developed countries^1^. According to recent reports, in 2016, over 22,280 new cases of epithelial ovarian cancer (EOC) would be identified, and over 14,240 deaths would occur in the United States^2^. The high mortality of this cancer is chiefly contributed to by the late diagnosis at stages III and IV, when the cancer cells are vigorously metastasizing, leading to a 5-year survival rate of approximately 21.9% and 5.6% for stages III and IV, respectively^3^. A better understanding of the mechanism of EOC metastasis is required to explore possible therapeutic strategies to increase the survival rate of ovarian cancer patients.

Mutations in p53 are found in more than 50% of human cancers, including lung, esophageal, colorectal, and ovarian cancers^4^. This tumor-associated alteration predominantly leads to a missense mutation located within the DNA-binding region, which has six “hotspot” amino acids that are the most frequently substituted (R175, G245, R248, R249, R273, and R282)^5^. Numerous studies have demonstrated that mutant p53 contributes to a more progressive tumor profile, suggesting that mutant p53 gains novel functions in promoting tumorigenesis and progression^6, 7^. In EOC, especially in the high-grade serous subtype, mutations in the TP53 gene are the most common and frequent events. According to The Cancer Genome Atlas project, TP53 was mutated in 96% of high-grade serous ovarian cancer (HGSOCs) samples^8^. In addition, most p53 mutations in ovarian cancer are missense mutations that are found in the DNA-binding domain with the hotspot codons R175, R248, and R273 (http://www-p53.iarc.fr/). Despite the prevalence of p53 mutations in ovarian cancer and the accumulating evidence for gain-of-function (GOF) cancer-associated p53 mutations, the role of p53 mutations in ovarian cancer and its underlying mechanisms are poorly understood.

Recent studies revealed that mutant p53 has binding partners, including NF-Y, ETS1, ETS2, p73, and p63, and it gains new oncogenic activities in tumor cells through interactions with these binding proteins^9–11^. For example, mutant p53 has been reported to interact with p63 and p73, p53 family proteins and transcription factors, and inhibit their transcriptional activity^9, 12^. In addition, the recruitment of mutant p53 to NF-Y-binding elements enhances proliferation in response to DNA-damaging agents^10^. Cohesins, multisubunit protein complexes, are highly conserved and play canonical roles in processes such as chromatin regulation, chromosome segregation, and DNA-damage response^13^. In addition to the canonical functions, recent studies have suggested a potential role of cohesins in cancer^13, 14^. Rad21 (double-strand-break repair protein rad21 homolog, also known as SCC1) is a component of the cohesin complex, which is crucial for chromosome segregation and DNA repair^14^. Interestingly, Rad21 has been shown to be co-localized with estrogen receptor α and associated with tumor progression in breast cancer cells^15^. However, little is known about the roles of Rad21 in tumor progression. Here, we identified Rad21 as a possible binding protein of mutant p53, which promotes cell invasion by regulating the transcriptional activity of Rad21 toward a subset of its target genes, in human ovarian cancer cells.

## Results

### The effect of mutant p53 expression on cell migration and invasion in p53-null ovarian cancer cells

**Figure 1:**
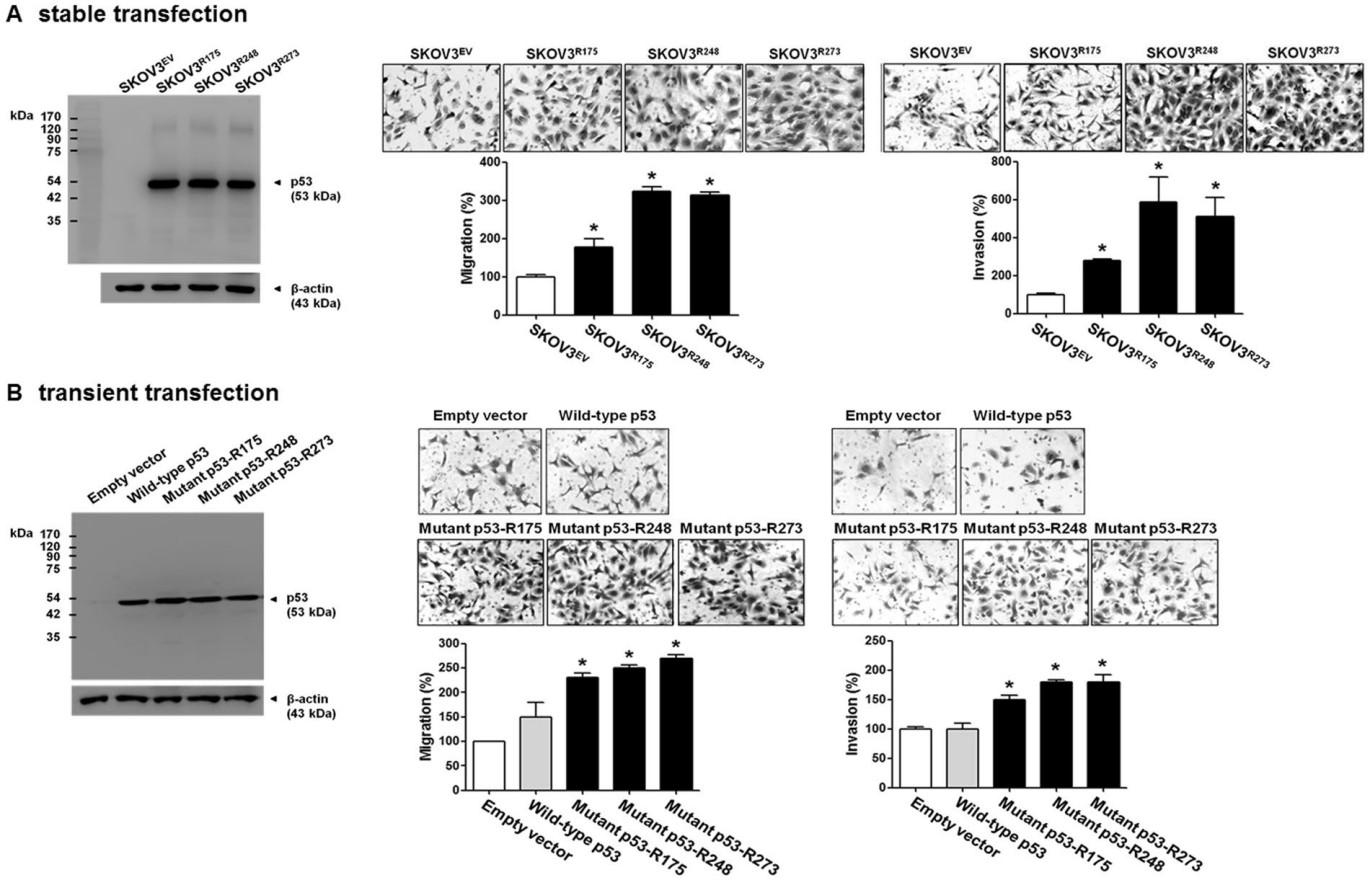
The effect of ectopic expression of p53 mutants on cell migration and invasion in p53-null SKOV3 cells. (**A**) A western blot assay was performed to measure the p53 protein levels in stably transfected SKOV3^EV^, SKOV3^R175^, SKOV3^R248^, and SKOV3^R273^ cells. β-Actin was used as an internal control. The cells were seeded in uncoated chambers for the migration assay and incubated for 24 h or seeded in Matrigel-coated chambers for the invasion assay and incubated for 48 h. (**B**) A western blot assay was performed to measure the p53 protein levels after SKOV3 cells were transiently transfected with empty vector, wild-type p53, p53-R175, p53-R248, and p53-R273. β-Actin was used as an internal control. The SKOV3 cells were seeded in uncoated chambers for the migration assay and incubated for 24 h or seeded in Matrigel-coated chambers for the invasion assay and incubated for 48 h. Representative images show the migrating and invading cells. Results are the combined data (mean ± S.D.) from three independent experiments. *P < 0.05 compared with the SKOV3^EV^ group or the empty vector group.

**Figure 2:**
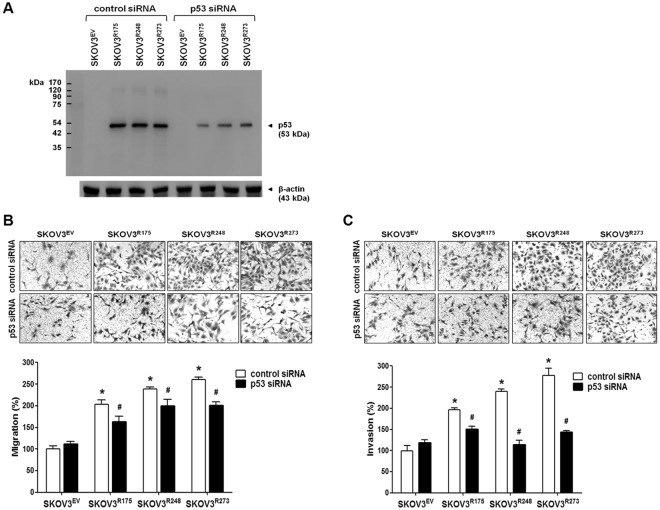
The effect of mutant p53 knockdown on mutant p53-induced ovarian cancer cell migration and invasion. (**A**) Stably transfected SKOV3^EV^, SKOV3^R175^, SKOV3^R248^, and SKOV3^R273^ cells were transfected with control or p53 siRNA, and then p53 protein levels were measured by western blot assay. β-Actin was used as an internal control. (**B**) After transfection with the siRNAs, the cells were seeded in uncoated chambers for the migration assay and incubated for 24 h. (**C**) After transfection with the siRNAs, the cells were seeded in Matrigel-coated chambers for the invasion assay and incubated for 48 h. Representative images show the migrating (**B**) or invading (**C**) cells. Results are the combined data (mean ± S.D.) from three independent experiments. *P < 0.05 compared with the control siRNA-transfected SKOV3^EV^ group and ^#^P < 0.05 compared with control siRNA-transfected SKOV3^R175^, SKOV3^R248^, and SKOV3^R273^ group, respectively.

We examined whether p53 mutants with a change at codons 175, 248, and 273 (p53-R175, p53-R248, and p53-R273, respectively), which are the hotspots for ovarian cancer-associated p53 missense mutations, are associated with the migration and invasion of ovarian cancer cells. SKOV3^R175^, SKOV3^R248^, and SKOV3^R273^, which were established through the stable transfection of p53-null SKOV3 cells with the p53-R175, p53-R248, and p53-R273 mutants, respectively, displayed more migration and invasion (Fig. 1A). Similarly, transient transfection of SKOV3 cells with the p53-R175, p53-R248, and p53-R273 mutants also displayed increased migration and invasion in p53-null SKOV3 cells (Fig. 1B). Among the mutants, the p53-R175 mutant seemed less effective than the other two mutants. To confirm the impact of p53 mutants on SKOV3 cell migration and invasion, we investigated the effect of p53 siRNA on the mutant p53-induced migration and invasion. As shown in Fig. 2, knockdown of the p53 mutants significantly attenuated the p53 mutant-induced migration and invasion in SKOV3 cells.

### The effect of mutant p53 on the expression of migration/invasion-associated genes in ovarian cancer cells

**Figure 3:**
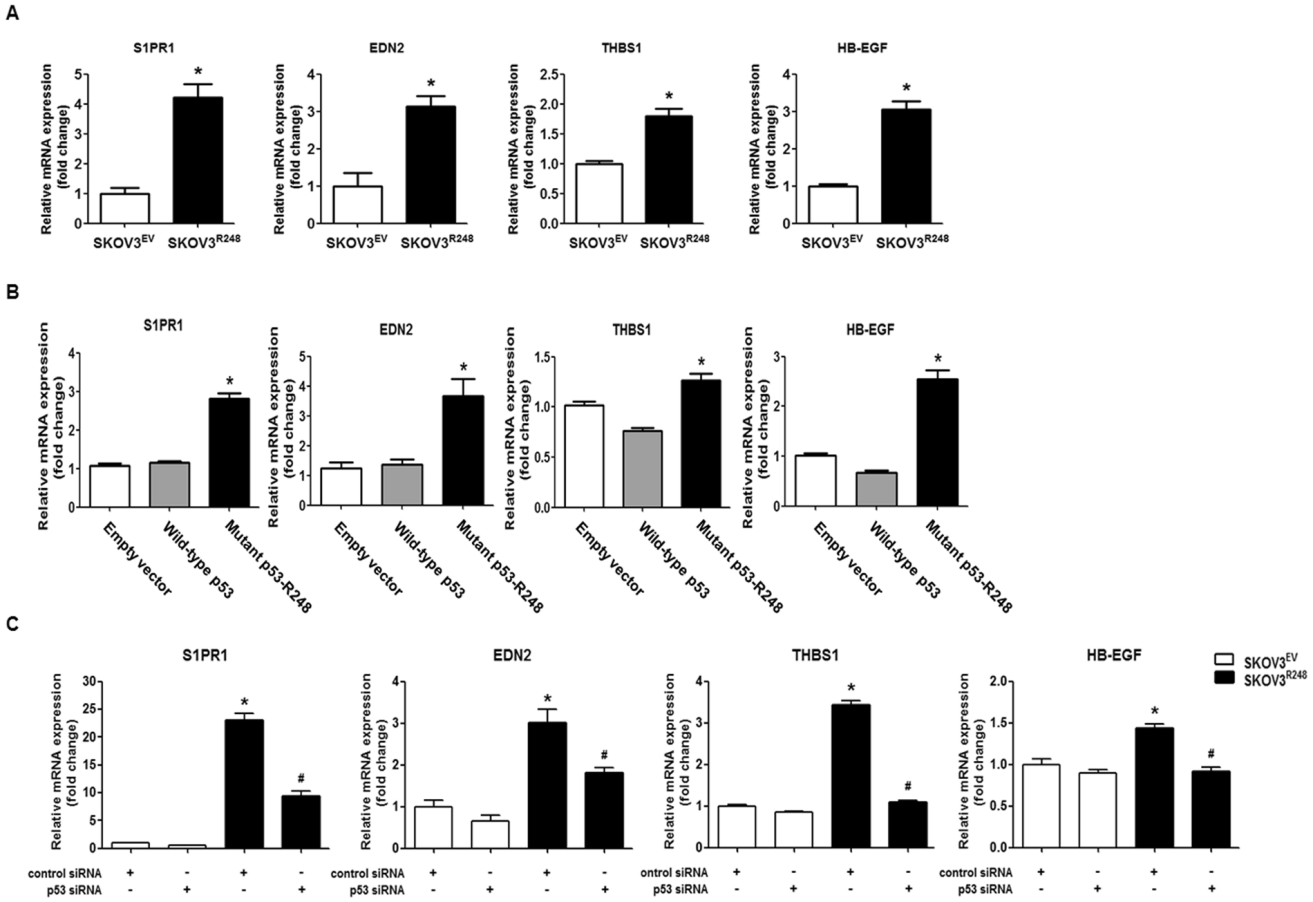
The effect of mutant p53 on the expression of migration/invasion-associated genes in ovarian cancer cells. (**A**) Real-time RT-PCR shows the mRNA levels of four selected genes (S1PR1, EDN2, THBS1, and HB-EGF) in stably transfected SKOV3^EV^ and SKOV3^R248^ cells. *P < 0.05 compared with the SKOV3^EV^ group. (**B**) SKOV3 cells were transiently transfected with empty vector, wild-type p53, and p53-R248. Real-time RT-PCR was performed to evaluate the mRNA levels of four selected genes (S1PR1, EDN2, THBS1, and HB-EGF) in the transiently transfected SKOV3 cells. *P < 0.05 compared with the empty vector group. (**C**) After transfection with p53 siRNA or control siRNA, real-time RT-PCR was performed to measure the mRNA levels of S1PR1, EDN2, THBS1, and HB-EGF in stably transfected SKOV3^EV^ and SKOV3^R248^ cells. *P < 0.05 compared with the control siRNA-transfected SKOV3^EV^ group and ^#^P < 0.05 compared with control siRNA-transfected SKOV3^R248^ group. All expression levels were normalized to GAPDH. Results are the combined data (mean ± S.D.) from three independent experiments.

To obtain insight into how mutant p53 mediates SKOV3 cell migration and invasion, we investigated the p53-R248-regulated gene expression through global transcript profiling by the Agilent Whole Human Genome Microarray. The results of the microarray analysis demonstrated that the expressions of 2,737 genes were significantly changed by mutant p53-R248 with a 1.5-fold increase or decrease (data not shown). By applying DAVID analysis to the 2,737 genes, the ontology group “positive regulation of cell migration”, which contains 27 migration/invasion-related genes, has been identified (Supplementary Table S2). Of the 27 genes, the top 4 genes (S1PR1, EDN2, THBS1, and HB-EGF) were selected for further studies. To validate the microarray data, we measured the mRNA levels of S1PR1, EDN2, THBS1, and HB-EGF using real-time RT-PCR. The stably transfected SKOV3^R248^ cells showed increased levels of S1PR1, EDN2, THBS1, and HB-EGF compared with SKOV3^EV^ cells (Fig. 3A). Similarly, transient transfection of SKOV3 cells with mutant p53-R248 resulted in a significant increase in the levels of the genes (Fig. 3B). These data demonstrated that ectopic expression of mutant p53 increases the expression of the migration/invasion-related genes, including S1PR1, EDN2, THBS1, and HB-EGF, in ovarian cancer cells. We further investigated whether the mutant p53 is responsible for the upregulation of S1PR1, EDN2, THBS1, and HB-EGF. As shown in Fig. 3C, the mRNA levels of a group of genes were significantly inhibited following knockdown of p53 in SKOV3^R248^ cells. These data suggested that modulation of S1PR1, EDN2, THBS1, and HB-EGF by mutant p53 may play a role in the mutant p53-R248-induced migration and invasion of ovarian cancer cells.

### Involvement of Rad21 in mutant p53-induced ovarian cancer cell invasion

**Figure 4:**
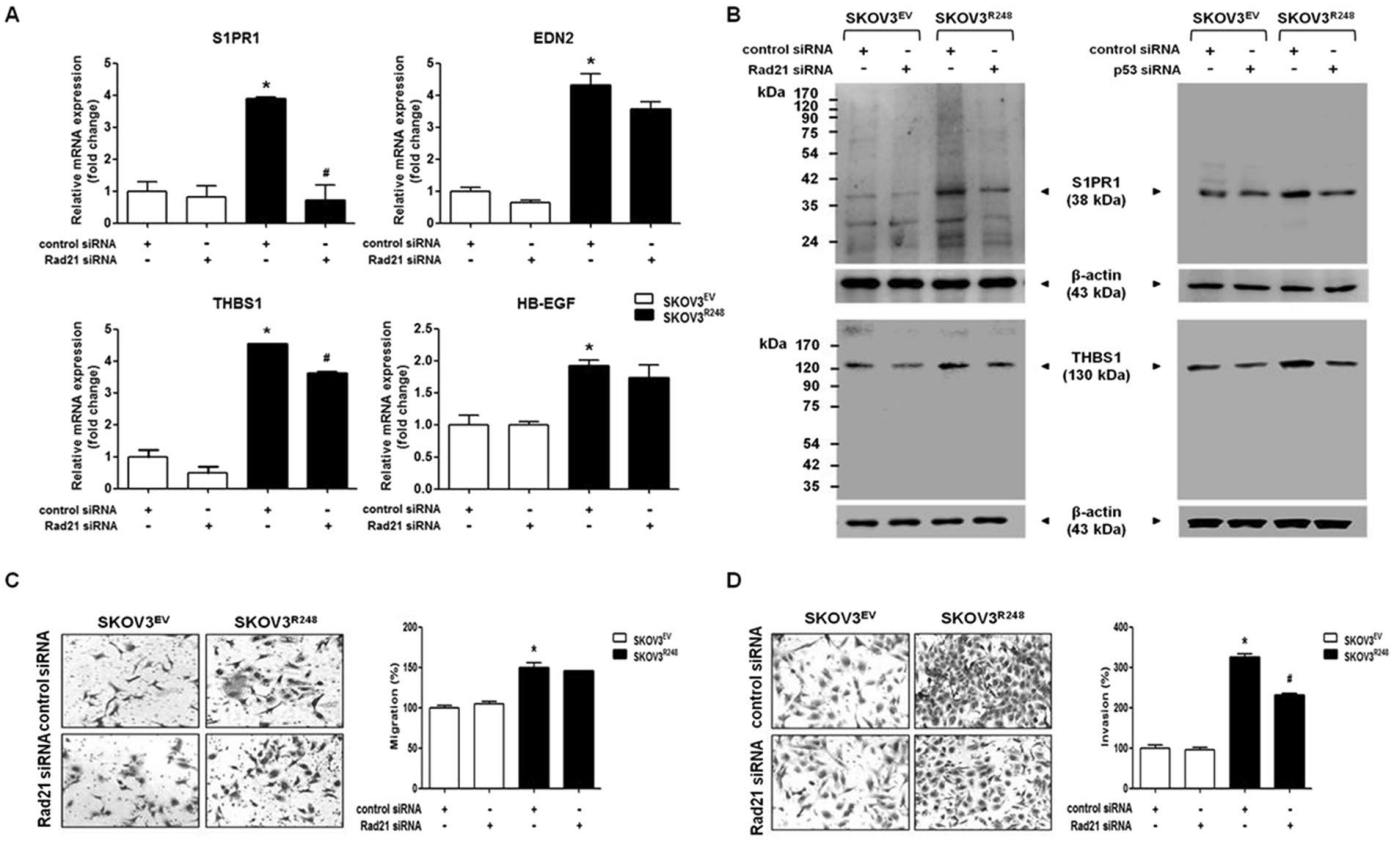
Involvement of Rad21 in mutant p53-induced ovarian cancer cell invasion. (**A**) The mRNA levels of S1PR1, EDN2, THBS1, and HB-EGF were measured by real-time RT-PCR after transfection with control siRNA or Rad21 siRNA in SKOV3^EV^ and SKOV3^R248^ cells. All expression levels were normalized to GAPDH. (**B**) The protein levels of S1PR1 and THBS1 were determined by western blot analysis after transfection with Rad21 siRNA (left panel) and p53 siRNA (right panel) in SKOV3^EV^ and SKOV3^R248^ cells. β-Actin was used as an internal control. (**C**) After transfection with siRNA, the cells were seeded in uncoated chambers for the migration assay and incubated for 24 h. (**D**) After transfection with siRNA, the cells were in Matrigel-coated chambers for the invasion assay and incubated for 48 h. Representative images show the migrating (**C**) and invading (**D**) cells. Results are the combined data (mean ± S.D.) from three independent experiments. *P < 0.05 compared with the control siRNA-transfected SKOV3^EV^ group and ^#^P < 0.05 compared with control siRNA-transfected SKOV3^R248^ group.

We assumed that mutant p53 interacts with a transcription factor to regulate the expression of all the four genes (S1PR1, EDN2, THBS1, and HB-EGF) in ovarian cancer cells. As described in the Methods, protein-binding sites enriched in each gene (S1PR1, EDN2, THBS1, and HB-EGF) were recognized, and then transcriptional regulators conserved in the transcription factor-binding sites for each gene were identified (Supplementary Table S3). We found that Rad21 is the only protein that has been identified as a potential transcription regulator for all four genes S1PR1, EDN2, THBS1, and HB-EGF, suggesting that Rad21 may be associated with transcriptional regulation of S1PR1, EDN2, THBS1, and HB-EGF induced by mutant p53. Because of this, we investigated the involvement of Rad21 in the expression of genes upregulated by mutant p53-R248. Knockdown of Rad21 inhibited the mutant p53-induced mRNA expression of S1PR1 and THBS1 but failed to show any significant change in EDN2 and HB-EGF mRNA expression (Fig. 4A). In addition, the mutant p53-induced protein expression of S1PR1 and THBS1 were also reduced by Rad21 knockdown as well as p53 knockdown (Fig. 4B). Next, we examined the role of Rad21 in cell migration and invasion induced by mutant p53. As shown in Fig. 4C and D, mutant p53-R248-induced invasion was partially inhibited by the downregulation of Rad21, whereas Rad21 downregulation did not affect the mutant p53-R248-induced migration. Taken together, our data suggested that Rad21 is mainly involved in cell invasion stimulated by mutant p53, but not migration, by transcriptionally regulating S1PR1 and THBS1.

### The effect of the Rad21 and mutant p53-R248 interaction on the transcriptional activation of S1PR1 and THBS1 in ovarian cancer cells

**Figure 5:**
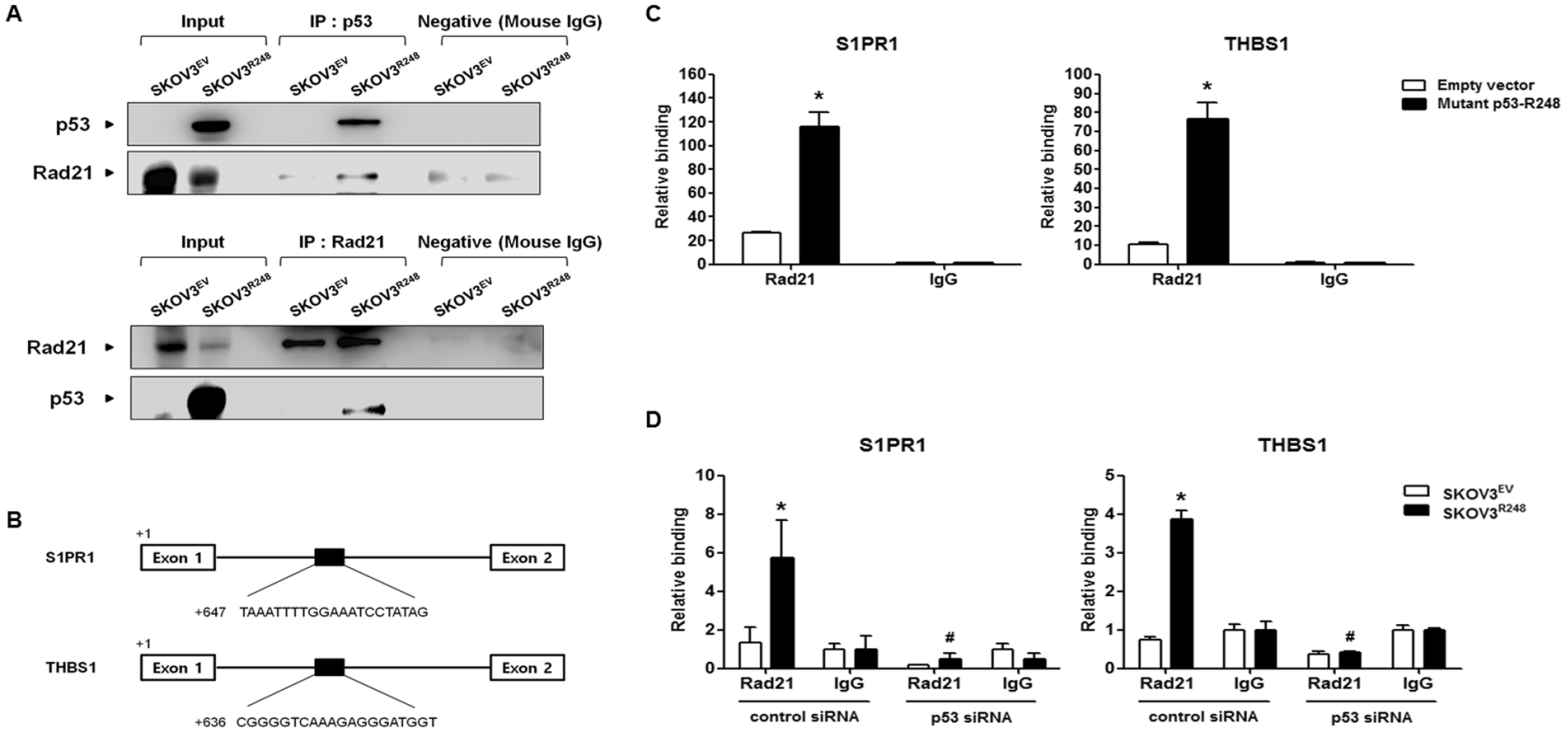
The effect of the mutant p53 and Rad21 interaction on the transcriptional activation of S1PR1 and THBS1 in ovarian cancer cells. (**A**) The protein interaction of Rad21 with mutant p53-R248 was determined by an immunoprecipitation assay in stably transfected SKOV3^EV^ and SKOV3^R248^ cells. The p53 protein was immunoprecipitated using an anti-p53 antibody. The reverse immunoprecipitation (IP) was also performed using an anti-Rad21 antibody. Proteins were analyzed by western blotting using anti-p53 and anti-Rad21-specific antibodies. Data are representative of three different experiments. (**B**) The putative Rad21-binding site in the S1PR1 and THBS1 genes. (**C**) After transient transfection with mutant p53-R248 or empty vector in SKOV3 cells, ChIP assays were performed. Cross-linked DNA fragments were immunoprecipitated with the anti-Rad21 antibody, and the purified DNA was amplified by PCR using gene-specific primers for the Rad21-binding sites of S1PR1 or THBS1. (**D**) After transfection with control siRNA or p53 siRNA in stably transfected SKOV3^EV^ and SKOV3^R248^ cells, respectively, ChIP assays were performed. Cross-linked DNA fragments were immunoprecipitated with the anti-Rad21 antibody, and the purified DNA was amplified by PCR using gene-specific primers for the Rad21-binding sites of S1PR1 or THBS1. The binding site was not detected when normal IgG was used or the antibody was omitted from the immunoprecipitation (IP) step. Data are representative of three different experiments. *P < 0.05 compared with the empty vector group or the SKOV3^EV^ group and ^#^P < 0.05 compared with control siRNA transfected-SKOV3^R248^ group.

Increasing evidence has demonstrated that mutant p53 can bind with other transcription factors and be recruited to their binding sites to regulate the expression of their target genes^16^. Thus, we investigated whether the interaction between mutant p53-R248 and Rad21 could enhance the ability of Rad21 to positively regulate the expression of S1PR1 and THBS1. We first examined the interaction between mutant p53-R248 and Rad21 by a co-IP assay. As shown Fig. 5A, binding of mutant p53-R248 with Rad21 was found in SKOV3^R248^ cells. We then performed a ChIP assay to elucidate whether the interaction of mutant p53 with Rad21 affects its binding to the transcription factor-binding site of the S1PR1 and THBS1 genes. Figure 5B shows putative Rad21-binding sites in the S1PR1 and THBS1 genes. Ectopic expression of mutant p53 significantly enhanced the binding of Rad21 to the binding sites (Fig. 5C). In addition, knockdown of mutant p53 markedly decreased the Rad21 binding in SKOV^R248^ cells (Fig. 5D). These observations suggested that mutant p53-R248 interacts with Rad21 and that this interaction may increase the transcription of migration/invasion-related genes, including S1PR1 and THBS1, in ovarian cancer cells.

### Involvement of S1PR1 in p53-R248-induced ovarian cancer cell invasion

**Figure 6:**
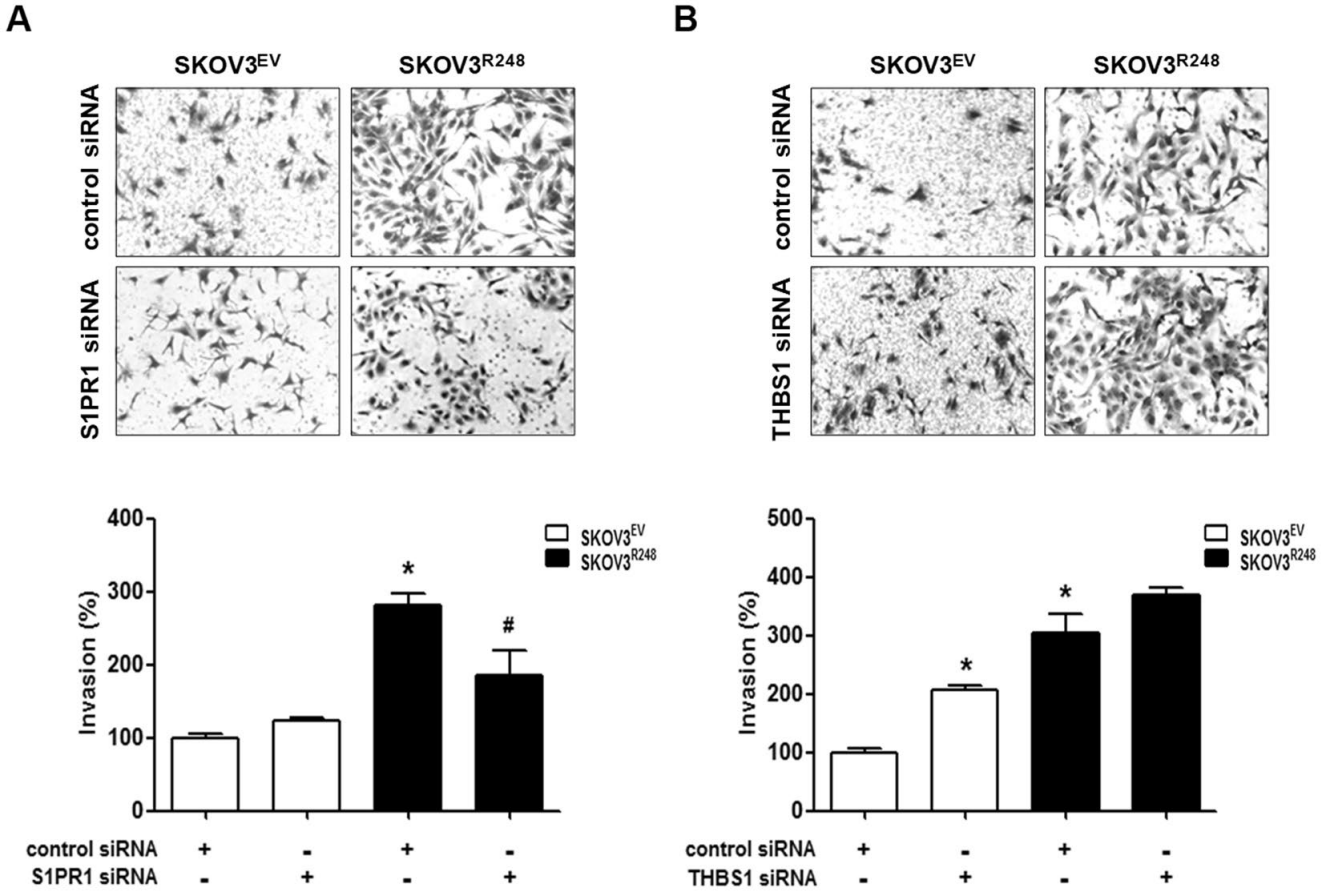
Involvement of S1PR1 in mutant p53-induced ovarian cancer cell invasion. (**A**) After transfection with control siRNA or S1PR1 siRNA for 24 h, the cells were seeded in Matrigel-coated chambers for invasion assay and incubated for 48 h. (**B**) After transfection with control siRNA or THBS1 siRNA for 24 h, the cells were seeded in Matrigel-coated chambers for invasion assay and incubated for 48 h. Representative images show the invading cells. Results are the combined data (mean ± S.D.) from three independent experiments. *P < 0.05 compared with the control-siRNA transfected SKOV3^EV^ group and ^#^P < 0.05 compared with control siRNA-transfected SKOV3^R248^ group.

We further tested the possibility that S1PR1 and/or THBS1 could mediate the promoting effect of Rad21 on mutant p53-induced invasion. Knockdown of S1PR1 using siRNA significantly reduced the invasive ability of SKOV3^R248^ cells (Fig. 6A), while THBS1 siRNA did not induce any significant change in cell invasion (Fig. 6B). Taken together, these results suggested that S1PR1, but not THBS1, plays a key role in the mutant p53-R248-induced ovarian cancer cell invasion.

## Discussion

Ovarian cancer is a highly metastatic cancer and has the highest mortality rate of all gynecologic cancers. Over 70% of ovarian cancer patients are diagnosed when the cancer has spread beyond the ovary to the peritoneum and omentum (stage III) and even to the liver and lung (stage IV). Despite current medical and technological advances, highly aggressive metastasis is still responsible for a poor survival rate of patients with ovarian cancer. At the new metastatic sites, cancer cells interact with the new microenvironment leading to the establishment of cancer cell migration and invasion. Due to the complex process, numerous molecules seem to be involved in the migration and invasion of ovarian cancer cells, resulting in the resistance to conventional therapies. Therefore, understanding the pathogenesis of ovarian cancer metastasis at the systemic, cellular, and molecular levels is important for the development and evaluation of new approaches to ovarian cancer treatment.

In addition to the loss of tumor suppressor function of wild-type p53, emerging evidence suggests that the common types of cancer-associated p53 mutations, with only a single amino acid substitution, lead to overexpression of the full-length p53 protein with new activities that can contribute to the development and progression of cancer^6, 7^. Indeed, mutant p53 has been suggested to be associated with proliferation, survival, angiogenesis, drug resistance, and genomic instability in cancer cells^17–21^. In particular, the importance of metastasis as a determinant of survival in most cancers has attracted many researchers to study the role of mutant p53 in cancer metastasis. For example, Noll, J.E. *et al*. suggested that mutant p53-expressing breast cancer cells showed a change in morphology and enhanced invasive capabilities^22^. Wang H. *et al*. showed that mutant p53 promotes migration and invasion abilities in endometrial cancer cells^23^. In addition, mutant p53 enhanced invasion in colon carcinomas through hepatocyte growth factor (HGF)-induced mesenchymal–epithelial transition (MET) signaling^24^. In transgenic mice models, mutations in p53 induced severe tumor phenotypes, including invasive carcinoma and frequent pulmonary metastasis^25^. With regard to ovarian cancer, a recent *in vivo* study demonstrated that ovarian tumors homozygous for a p53 mutation were undifferentiated and highly metastatic, and expressed genes with potential regulatory functions in EOC^26^. In addition, p53 mutations in ovarian cancer patients are significantly associated with a higher incidence of platinum resistance, local recurrence, and distant metastasis^27^. However, the concrete molecular mechanisms underlying the mutant p53 GOF for ovarian cancer metastasis have been poorly elucidated. In a previous study, we demonstrated that overexpressing mutant p53 enhanced the ability of ovarian cancer cells to adhere to mesothelial cells via integrin β4 and the Akt pathway^28^. In addition, Iwanicki *et al*. revealed that mutant p53 enhanced an anchorage independence and the subsequent mesothelial intercalation in the fallopian tube nonciliated epithelial (FNE) cells via fibronectin production, α5β1 fibronectin receptor engagement, and TWIST1 expression^29^. These data suggested that mutant p53 plays a pivotal role in ovarian cancer cell adhesion to mesothelial cells, which is the initial step for ovarian cancer metastasis. Here, our data suggested for the first time that mutant p53 is involved in the migration and invasion of ovarian cancer cells, as well as their adhesion. Ovarian cancer cells expressing p53 mutants acquired migratory and invasive capacity, and this capacity was clearly reduced by knockdown of mutant p53. Taken together, mutant p53 seems to increase aggressiveness and higher metastatic potential of ovarian cancer cells, providing the relevance for previous *in vivo* and clinical studies in ovarian cancer metastasis.

Similar to the function of wild-type p53 as a transcription factor, mutant p53 has been suggested to exert its function by transcriptionally regulating its target genes^30^. Most p53 mutations generate stable full-length proteins with the substitution of a single amino acid (stable variant proteins), leading to the loss of the ability to bind to canonical p53 consensus sequences. Instead of direct transcriptional regulation by binding to canonical p53 consensus elements, it has been reported that mutant p53 interacts with non-typical transcription factors and can be recruited to their regulative elements, which contribute to the up- or downregulation of their target gene expression to induce GOF mutant p53. For example, mutant p53 has been suggested to regulate the expression of a variety of genes associated with chemoresistance, including c-Myc, CXCL1, PCNA, ABCB1 (MDR1), and IGFIR^31–36^, and with proliferation and anti-apoptosis, including c-Myc, CXCL1, MAPK family genes, CCNA2, CCNB1, CCNB2, NF-kB, and ABCB1 (MDR1)^31–33, 37–39^. In addition, IGF1R, PXN, Twist, SHARP1, and CCNG2, which are associated with migration and invasion, have been reported to be regulated by mutant p53^11, 33, 40^. In the present study, in order to elucidate the molecular mechanisms of mutant p53-induced migration and invasion of ovarian cancer cells, we performed global gene expression analysis of ovarian cancer cells expressing mutant p53 and identified 27 migration/invasion-associated genes including S1PR1, EDN2, THBS1, and HB-EGF. In addition, S1PR1 has been shown to be associated with the mutant p53-induced invasion of ovarian cancer cells.

S1PR1 (Sphingosine 1-phosphate receptor 1), a G-protein-coupled receptor, binds to the bioactive lysosphingolipid sphingosine 1-phosphate (S1P), which modulates diverse cellular processes, including cell proliferation, adhesion, migration, invasion, and angiogenesis^41–45^. Several studies have demonstrated that SP1/S1PR1 modulated cell growth, adhesion, migration, and invasion in ovarian cancer cells^46–49^. For example, activated S1PR1 leads to the migration of ovarian cancer cells via PKC and RhoA signaling^47^. Park *et al*. have suggested that S1P increased the migration and invasion of S1PR1-expressing ovarian cancer cells through the Akt and p38 MAPK pathway^48^. Although it has been suggested that S1P, a ligand for S1PR1, is aberrantly produced in ovarian cancer patients and that p53 is mutated in most ovarian cancer patients, little is known about the interaction between S1PR1 and the mutant p53 in ovarian cancer. This study provides the first evidence to show that overexpression of mutant p53 in ovarian cancer can regulate S1PR1 expression, resulting in an increase in ovarian cancer invasion. However, the detailed molecular mechanisms by which S1PR1 mediates the mutant p53-induced invasion in ovarian cancer cells should be further investigated.

In the current study, Rad21 was identified as a new binding partner of mutant p53. Rad21 is a double-strand-break repair protein and a component of the cohesin complex, which plays a crucial role in chromosome segregation and DNA repair^13^. Interestingly, recent studies have reported that Rad21 plays a role in several cancers. For example, knockdown of Rad21 significantly inhibited the proliferation of breast cancer MCF-7 cells and increased their sensitivity to respond to chemotherapeutic the agents etoposide and bleomycin, suggesting that Rad21 is associated with proliferation and chemoresistance in breast cancer cells^50^. In addition, overexpression of Rad21 was found in the group of breast cancer patients with supraclavicular lymph node metastases, which had poor prognosis^51, 52^. Interestingly, Rad21 was found to be overexpressed in undifferentiated ovarian cancer^53^. However, the role of Rad21 in ovarian cancer development and progression has yet to be elucidated. In this study, we have demonstrated that Rad21 interacts with mutant p53 to increase the transcriptional activity of S1PR1, resulting in the enhanced invasion of ovarian cancer cells. Interestingly, several studies have shown that Rad21 co-localizes with transcription factors, such as estrogen receptor α in breast cancer cells and the mediator complex, which plays a key role in transcription^15, 54, 55^. In addition, Rad21 was shown to regulate the expression of Runx1, which is associated with cell differentiation, growth, and survival^56^. The detailed molecular mechanism by which Rad21 regulates the expression of mutant p53-targeted genes such as S1PR1 remains to be investigated. Notably, Rad21 knockdown showed only partial reverse of the mutant p53-induced invasion in SKOV3 cells, suggesting that other migration/invasion molecules may play a role in mutant p53-enhanced invasion in ovarian cancer cells. Recent studies suggested that mutant p53 regulates several migration/invasion genes such as Twist1 and Sharp1 in other types of cancer cells^11, 40^. Further experimentation is required to investigate the potential role of the genes in the mutant p53-enhanced ovarian cancer cell invasion.

## Material and Methods

### Material

RPMI1640 medium, Opti-modified Eagle’s medium (Opti-MEM), fetal bovine serum (FBS), penicillin, and streptomycin were purchased from Life Technologies, Inc. (Grand Island, NY, USA). Dimethyl sulfoxide (DMSO), RNase A, leupeptin, aprotinin, phenylmethylsulfonylfluoride, and Triton X-100 were purchased from Sigma-Aldrich Co. (St Louis, MO, USA). The pCMV-Neo-Bam p53-R175H, pCMV-Neo-Bam p53-R248W, pCMV-Neo-Bam p53-R273H, and pCMV-Neo-Bam p53 wild-type were obtained from Addgene (Cambridge, MA, USA). The antibodies against anti-p53, anti-Rad21, anti-S1PR1, anti-THBS1, and anti-β-actin were purchased from Santa Cruz Biotechnology (Santa Cruz, CA, USA). The protein G PLUS-agarose was also obtained from Santa Cruz Biotechnology (Santa Cruz, CA, USA).

### Cell culture and transfection

The high grade serous human ovarian cancer cell line SKOV3 was originally from the American Type Culture Collection. The cells were maintained in RPMI1640 with 5% FBS, 100 U/ml penicillin, and 100 μg/ml streptomycin sulfate in a humidified atmosphere of 5% CO_2_–95% air at 37 °C. As described in our previous study, cells were plated in 6-well culture dishes and allowed to attach and grow for 24 h before transfection. Each transfection mixture was prepared by mixing the 1 μg DNA and 5 μg/ml polyethylenimine (BD Biosciences, San Joes, CA, USA) in serum-free Opti-MEM and incubating for 15 min at room temperature. The transfection mixture was slowly added to the cells, which were allowed to recover for an additional 24 h before experiment. Stable clones (SKOV3^EV^, SKOV3^R175^, SKOV3^R248^, and SKOV3^R273^) were selected in the presence of 200 μg/ml G418 (Sigma-Aldrich Co., St Louis, MO, USA) and expanded, and gene expression was confirmed by western blotting^28^.

### Gene knockdown using siRNA

Small interfering RNAs (siRNAs) for p53, Rad21, S1PR1, THBS1, and control siRNA were purchased by Bioneer technology (Daejon, South Korea). Cells were transfected with siRNA at a final concentration of 50 nM using lipofectamine (Invitrogen, Carlsbad, CA, USA), according to the manufacturer’s instructions. Briefly, cells were plated in 6-well culture dishes and allowed to attach and grow for 24 h before transfection. Each transfection mixture was prepared by mixing the siRNA and lipofectamine in serum-free Opti-MEM and incubating for 10 min at room temperature. The transfection mixture was slowly added to the cells, which were allowed to recover for an additional 24 h before experiment. The silencing efficiency of siRNAs was clearly shown in Fig. 2A (p53 siRNA) and Supplementary Figure S1 (Rad21 siRNA) and Figure S2 (S1PR1 and THBS1).

### RNA isolation and real-time RT–PCR analysis

Total RNA was prepared the Easy Blue® kits (Intron Biotechnology, Seoul, South Korea), according to the manufacturer’s instructions. Total RNA was reverse transcribed into first-strand cDNA (Amersham Pharmacia Biotech, Piscataway, NJ, USA) following the manufacturer’s instructions. The synthesized cDNA was used as a template for polymerase chain reaction (PCR) amplification. Real-time RT-PCR was performed using Thermal Cycler Dice Real-time PCR System (Takara, Tokyo, Japan). The primers used for SYBR Green real-time RT-PCR are listed in Supplementary Table S1. A dissociation curve analysis of p53, Rad21, S1PR1, EDN2, THBS1, HB-EGF, and GAPDH showed a single peak. PCRs were carried out for 50 cycles using the following conditions: denaturation at 95 °C for 5 s, annealing at 55 °C for 10 s, and elongation at 72 °C for 20 s. The results for p53, Rad21, S1PR1, EDN2, THBS1, and HB-EGF mRNA are normalized to a control gene GAPDH.

### Western blot analysis

The cells were washed with ice-cold phosphate-buffered saline (PBS) and extracted by protein lysis buffer (Intron Biotechnology, Seoul, South Korea). The protein concentration was determined by the Bradford assay. The cell lysates were mixed with 5× sodium dodecyl sulfate (SDS) sample buffer, boiled for 5 min, and then separated on 10% SDS-PAGE. After electrophoresis, the proteins were transferred to polyvinylidene difluoride (PVDF) membranes. The membrane was blocked in 5% nonfat dry milk for 30 min, rinsed and incubated with specific antibodies against p53 (mouse monoclonal, 1:2500), Rad21 (mouse monoclonal, 1:500), S1PR1 (mouse monoclonal, 1:1000), THBS1 (mouse monoclonal, 1:000), and β-actin (mouse monoclonal, 1:5000) in Tris-buffered saline containing 5% nonfat dry milk and Tween-20 (0.1%) overnight at 4 °C (TBS-T). The membranes were washed three times to remove the primary antibodies and incubated for 2 h with a horseradish peroxidase-conjugated secondary antibody (1:1000–2500). After washing three times with TBS-T, immunopositive bands were visualized by enhanced chemiluminescence (ECL; Abclon, Seoul, South Korea) and analyzed using ImageQuant Las-4000 (GE Healthcare Life Science, Milwaukee, WI, USA).

### Transwell-migration assay

*In vitro* transwell-migration assay was performed using a transwell unit (8-µm pore size) with polyvinylpyrrolidone-free polycarbonate filters. The filters were washed thoroughly in PBS and dried immediately before use. After transfection with DNA plasmid or siRNA, trypsinized cells (1.5 × 10^4^) were resuspended in medium RPMI1640 containing 1% FBS and added to the top chamber. Culture medium containing 5% FBS was then added to the bottom chamber. The cells were incubated at 37 °C and allowed to migrate to the lower surface of the membrane for 24 h. Following incubation, filters were fixed with methanol for 10 min and stained with 0.5% crystal violet (BD Biosciences, San Joes, CA, USA) for 30 min. Non- migratory cells were removed using a cotton swab, whereas migratory cells on the underside of the filter were counted using an inverted microscope. All experiments were done in triplicate, and a minimum of five fields per filter were counted.

### Invasion assay

*In vitro* cellular invasion was assayed by determining the ability of cells to invade a synthetic basement membrane (Matrigel, Corning, Steuben County, NY, USA). Briefly, polycarbonate filters (8-µm pore size) were coated with Matrigel at a concentration of 1 µg/ml and placed in a modified Boyden chamber. After transfection with DNA plasmid or siRNA, trypsinized cells (1.0 × 10^5^) were resuspended in medium RPMI1640 containing 1% FBS and added to the top chamber. Culture medium containing 5% FBS was then added to the bottom chamber. The cells were incubated at 37 °C and allowed to invade through the Matrigel barrier for 48 h. Following incubation, filters were fixed with methanol for 10 min and stained with 0.5% crystal violet for 30 min. Non-invading cells were removed using a cotton swab, whereas invading cells on the underside of the filter were counted using an inverted microscope. All experiments were done in triplicate, and a minimum of five fields per filter were counted.

### Immunoprecipitation (IP) assay

After harvesting and washing, cells were lysed by repeated aspiration through a 21 gauge needle in a high-salt buffer (50 mM Tris-HCl, 150 mM NaCl, 1% NP-40, pH 8.0, protease inhibitor) and incubated for 1 h on ice. After centrifugation (10,000 g, 5 min), protein concentrations were determined. Equal amount of protein (500 μg) was incubated with anti-p53 or anti-Rad21 antibodies for 1 h at 4 °C, followed by incubation with 20 μl protein G-sepharose beads overnight at 4 °C. The protein complex was washed 4 times and released from the beads by boiling in 2× sample buffer (350 mM Tris, pH 6.8, 10% SDS, 30% β-mercaptoethanol, 6% glycerol, 0.12% bromophenolblue) for 10 min. The eluted proteins were electrophoresed by 8–10% SDS-PAGE, transferred to PVDF membranes, and probed with anti-p53 or anti-Rad21 antibodies. The signals were visualized using an ECL chemiluminescent system. Following three washes in TBS-T, immunopositive bands were visualized by enhanced chemiluminescence and exposed to Image Quant LAS-4000.

### Chromatin immunoprecipitation (ChIP) assay

Chromatin immunoprecipitation (ChIP) was performed using a ChIP Assay Kit, according to the manufacturer’s instructions (Upstate Biotechnology, Lake Placid, NY, USA). Cells were treated with formaldehyde (final concentration of 1%) for 10 min at 37 °C. Chromatin extracts were sonicated to obtain DNA fragments with size of 300–500 bp and then they were immunoprecipitated with anti-Rad21 antibody overnight at 4 °C. After they were pulled down with salmon sperm DNA-protein G-agarose beads, cross-links were reversed. DNA was purified using Fragment DNA purification kit (Intron Biotechnology, Seoul, South Korea), prior to its use in the PCR reaction. The immunoprecipitated sample DNA was compared with the input DNA. The primers used to amplify a distinct region of the S1PR1 and THBS1 with putative Rad21-binding sites were described in Supplementary Table S1.

### Statistical analysis

Statistical data are presented as the mean ± S.D. of three individual experiments performed in triplicate. Statistical analysis was carried out using Student’s t-test or a one-way ANOVA, and the level of significance was set at a P value of <0.05.

## Electronic supplementary material

Suppementary information

Figure S1
