# Author Correction: Mutant p53 stimulates cell invasion through an interaction with Rad21 in human ovarian cancer cells

**DOI:** 10.1038/s41598-018-29327-4

**Published:** 2018-07-18

**Authors:** Ji-Hye Ahn, Tae Jin Kim, Jae Ho Lee, Jung-Hye Choi

**Affiliations:** 1 Department of Life and Nanopharmaceutical Sciences, Kyung Hee University, Seoul, 02447 South Korea; 2 Division of Molecular Biology, College of Pharmacy, Kyung Hee University, Seoul, 02447 South Korea; 3 Department of Obstetrics and Gynecology, Cheil General Hospital and Women’s Healthcare Center, Dankook University College of Medicine, Seoul, 04619 South Korea; 4 Laboratory of Molecular Oncology, Cheil General Hospital and Women’s Healthcare Center, Dankook University College of Medicine, Seoul, 04619 South Korea

Correction to: *Scientific Reports* 10.1038/s41598-017-08880-4, published online 22 August 2017

The original version of this Article contained errors.

In Figure 1, where the photo showing the invasion of SKOV3 cells stably transfected with empty vector (SKOV3^EV^) in panel A was a duplication of the photo showing the migration of SKOV3 cells transiently transfected with empty vector in panel B, and the photos showing the migration and invasion of SKOV3 cells transfected with mutant-R273 in panels A and B were different version of photos showing that of SKOV3 cells transfected with mutant-R248.

**Figure 1:**
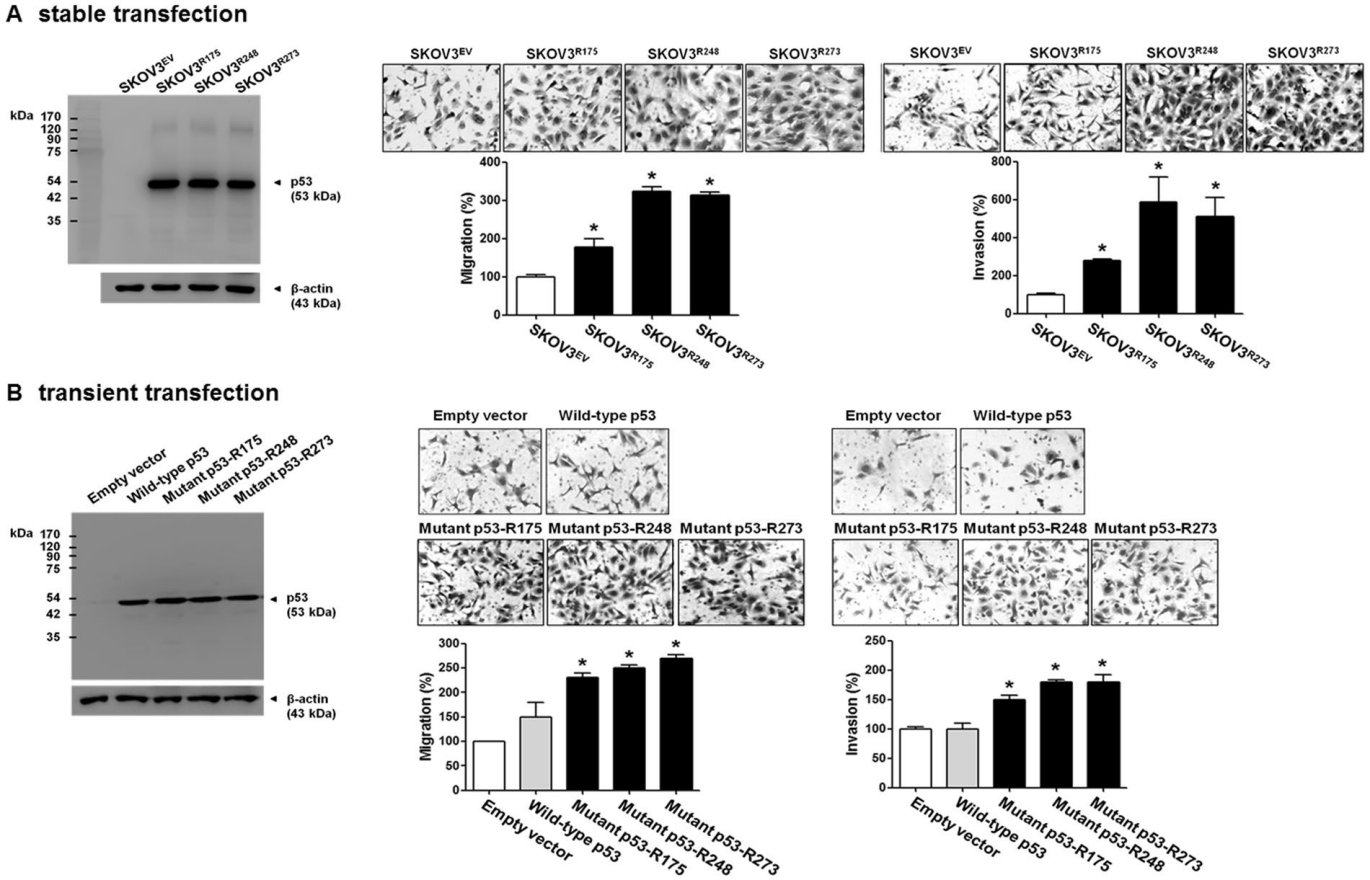
The effect of ectopic expression of p53 mutants on cell migration and invasion in p53-null SKOV3 cells. (**A**) A western blot assay was performed to measure the p53 protein levels in stably transfected SKOV3^EV^, SKOV3^R175^, SKOV3^R248^, and SKOV3^R273^ cells. β-Actin was used as an internal control. The cells were seeded in uncoated chambers for the migration assay and incubated for 24 h or seeded in Matrigel-coated chambers forthe invasion assay and incubated for 48 h. (**B**) A western blot assay was performed to measure the p53 protein levels after SKOV3 cells were transiently transfected with empty vector, wild-type p53, p53-R175, p53-R248, and p53-R273. β-Actin was used as an internal control. The SKOV3 cells were seeded in uncoated chambers for the migration assay and incubated for 24 h or seeded in Matrigel-coated chambers for the invasion assay and incubated for 48 h. Representative images show the migrating and invading cells. Results are the combined data (mean ± S.D.) from three independent experiments. *P < 0.05 compared with the SKOV3^EV^ group or the empty vector group.

In Figure 4, where the photo showing the migration of SKOV3^EV^ cells transfected with Rad21 siRNA in panel C was a duplication of the photo showing the migration of SKOV3^EV^ cells transfected with control siRNA.

**Figure 4:**
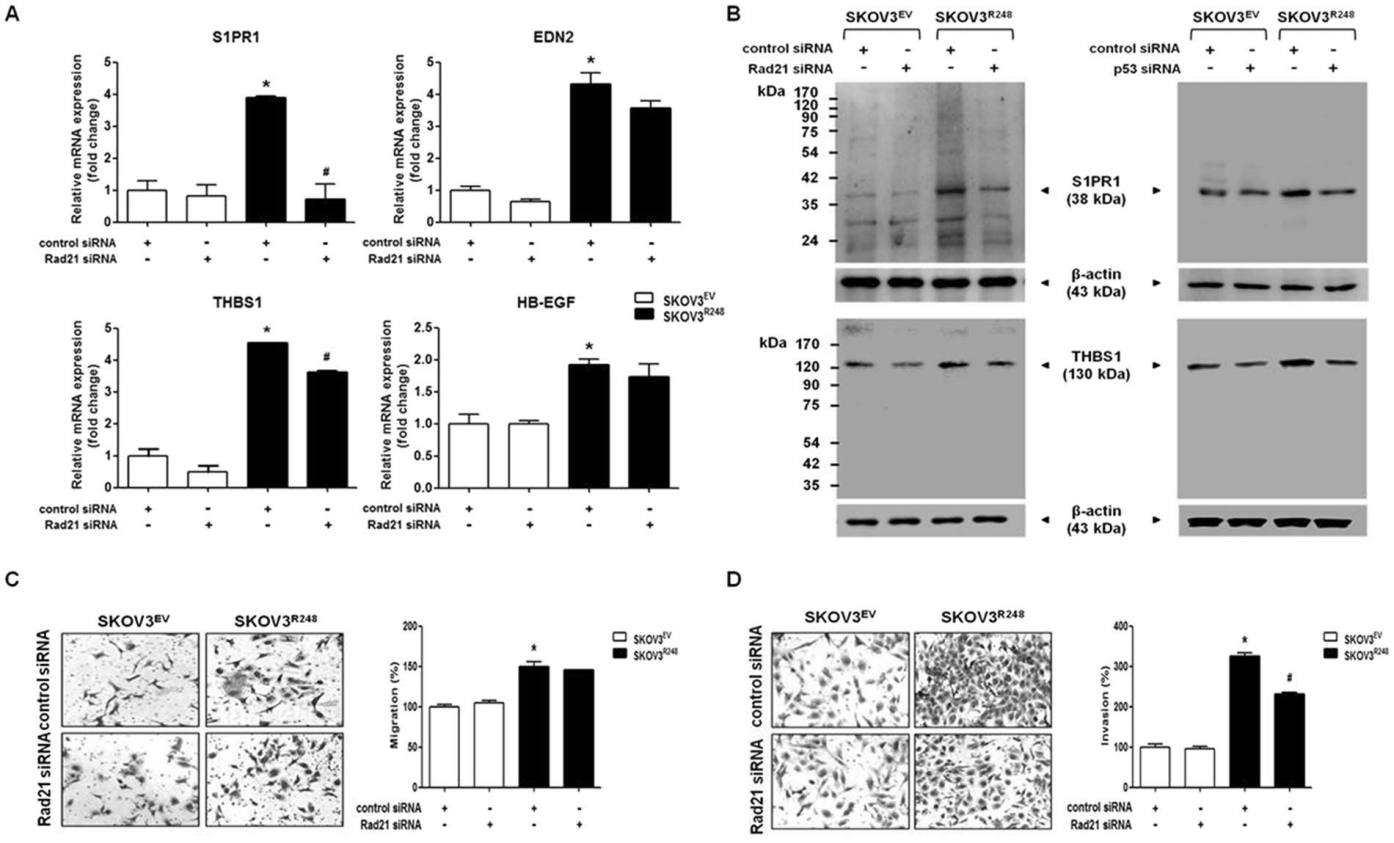
Involvement of Rad21 in mutant p53-induced ovarian cancer cell invasion. (**A**) The mRNA levels of S1PR1, EDN2, THBS1, and HB-EGF were measured by real-time RT-PCR after transfection with control siRNA or Rad21 siRNA in SKOV3^EV^ and SKOV3^R248^ cells. All expression levels were normalized to GAPDH. (**B**) The protein levels of S1PR1 and THBS1 were determined by western blot analysis after transfection with Rad21 siRNA (left panel) and p53 siRNA (right panel) in SKOV3^EV^ and SKOV3^R248^ cells. β-Actin was used as an internal control. (**C**) After transfection with siRNA, the cells were seeded in uncoated chambers for the migration assay and incubated for 24 h. (**D**) After transfection with siRNA, the cells were in Matrigel-coated chambers for the invasion assay and incubated for 48 h. Representative images show the migrating (**C**) and invading (**D**) cells. Results are the combined data (mean ± S.D.) from three independent experiments. *P < 0.05 compared with the control siRNA-transfected SKOV3^EV^ group and ^#^P < 0.05 compared with control siRNAtransfected SKOV3^R248^ group.

In Figure 6, where the photo showing the invasion of SKOV3^EV^ cells transfected with S1PR1 siRNA in panel A was a duplication of the photo showing the invasion of SKOV3^EV^ cells transfected with control siRNA.

**Figure 6:**
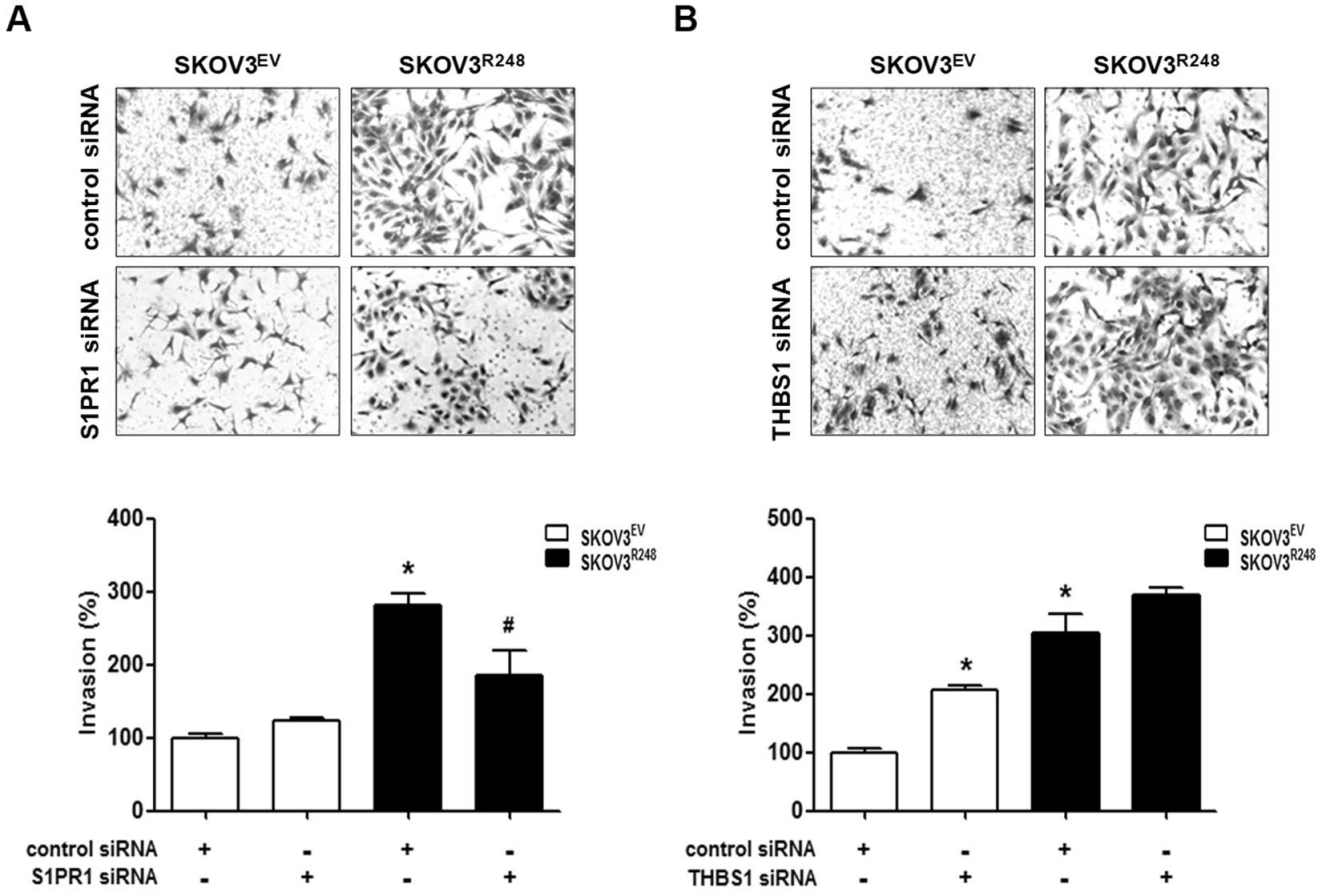
Involvement of S1PR1 in mutant p53-induced ovarian cancer cell invasion. (**A**) After transfection with control siRNA or S1PR1 siRNA for 24 h, the cells were seeded in Matrigel-coated chambers for invasion assay and incubated for 48 h. (**B**) After transfection with control siRNA or THBS1 siRNA for 24 h, the cells were seeded in Matrigel-coated chambers for invasion assay and incubated for 48 h. Representative images show the invading cells. Results are the combined data (mean ± S.D.) from three independent experiments. *P < 0.05 compared with the control-siRNA transfected SKOV3^EV^ group and ^#^P < 0.05 compared with control siRNAtransfected SKOV3^R248^ group.

These errors have been corrected in the HTML and PDF versions of the Article.

Additionally, images of the full immunoblots have been uploaded as a Supplementary Information file.

