# Supplementary material for: Mutant p53 stimulates cell invasion through an interaction with Rad21 in human ovarian cancer cells
Source: Sci Rep. 2017 Aug 22;7:9076. doi: 10.1038/s41598-017-08880-4 (PMC5567302; doi:10.1038/s41598-017-08880-4)

SUPPLEMENTARY INFORMATION

Mutant p53 stimulates cell invasion through an interaction with Rad21 in human ovarian cancer cells

Ji-Hye Ahn[1,2], Tae Jin Kim[3], Jae Ho Lee[4], and Jung-Hye Choi [1,2,*]

[1] Department of Life and Nanopharmaceutical Sciences, Kyung Hee University, Seoul 02447, South Korea

[2] Division of Molecular Biology, College of Pharmacy, Kyung Hee University, Seoul 02447, South Korea

[3] Department of Obstetrics and Gynecology, Cheil General Hospital and Women's Healthcare Center, Dankook University College of Medicine, Seoul 04619, South Korea

[4] Laboratory of Molecular Oncology, Cheil General Hospital and Women's Healthcare Center, Dankook University College of Medicine, Seoul 04619, South Korea

*Corresponding author

Jung-Hye Choi

Phone: +82-2-961-2172. Fax: +82-2-962-3885.

E-mail: jchoi@khu.ac.kr

Supplementary methods

Microarray analysis

The Agilent Whole Human Genome Microarray (Agilent, Foster City, CA, USA) was used to analyse the gene expression in SKOV3^{EV} and SKOV3^{R248} cells. RNA was prepared from the cells using Easy Blue® kits (Intron Biotechnology, Seoul, South Korea). Labelling, hybridisation, and scanning of the arrays were performed according to the manufacturer's instructions at E-biogen Inc. (Seoul, South Korea). To screen for transcripts differentially expressed in SKOV3^{EV} versus SKOV3^{R248} cells, transcripts with a fold change of > 1.5 were considered to be significantly regulated. The "Database for Annotation, Visualization, and Integrated Discovery" (DAVID) program was used for gene annotation. The "Functional Annotation Tool" in the online version of DAVID (http://david.abcc.ncifcrf.gov/) was run using the default parameters and focusing on the categories Gene-Ontology-Biological Process.

Transcriptional regulation and conservation data analysis

To search for predicted transcription factors-binding site, we used human Chip-seq data from the Encyclopedia of DNA Elements (ENCODE) projects [1]. Chip-seq data for 161 transcription factors with the binding motifs from Factor book repository was visualized using the UCSC genome browser (http://genome.ucsc.edu/cgi-bin/hgGateway) and inspected for the genes-binding element region [2,3]. From this data, we have listed each transcription regulators conserved in transcription factor-binding site of S1PR1, EDN2, THBS1, and HB-EGF (Supplementary Table S3).

Supplementary Table 1. Primer sequences for real-time RT-PCR analysis

Gene	Sense primer	Antisense primer
**PCR primers**		
TP53	TGT TTC CTG ACT CAG AGG GG	GAG CGT GCT TTC CAC GAC
Rad21	GGC ACT GTT ACC ACA AAC CTT TGG	GGG GAC ATT TGA ATG CTG ACT GGC
S1PR1	ACC AAG AAA TTC CAC CGA CC-	AGG GCC ACA AAC ATA CTC CC
HB-EGF	TGT TTT GAA AGC CCA AGG TG	GGA CCA GGA AAG CTA CAG GC
EDN2	CCT GCA GAC GTG TTC CAG AC	TTC CTT CCC AAT GTT CCT CC
THBS1	TTT GGC TAC CAG TCC AGC	AGA AAG GCC CGA GTA TCC CT
GAPDH	GAG TCA ACG GAT TTG GTC GT	TTG ATT TTG GAG GGA TCT CG
**ChIP primers**		
S1PR1	TGG TCG GAG GAA TAG GAG GGA A	AAC AAT CCC AGC AAC TCC GCG A
THBS1	TCG GCT CTT GTG CTT CCT GCT A	TGG GAT AGC CAG CAC CTC TTC CTT

Supplementary Table 2. List of 27 migration/invasion-associated genes.

The 2,737 genes found to be significantly changed by mutant p53-R248 with a 1.5-fold increase or decrease were classified by DAVID [http://david.abcc.ncifcrf.gov/] into Biological Process classes. The table shows the 27 genes in "positive regulation of cell migration" class and fold-change present in our microarray

No	Gene symbol	Gene description	List ID	Fold Change (in Microarray)
1	S1PR1	sphingosine-1-phosphate receptor 1	NM_001400	2.9
2	EDN2	endothelin 2	NM_001956	2.8
3	THBS1	thrombospondin 1	NM_003246	1.6
4	HB-EGF	heparin-binding EGF-like growth factor	NM_001945	1.5
5	MYO1F	myosin IF	NM_012335	0.7
6	IRS1	insulin receptor substrate 1	NM_005544	0.7
7	ADAM10	ADAM metallopeptidase domain 10	NM_001110	0.7
8	RPS6KB1	ribosomal protein S6 kinase, 70kDa, polypeptide 1	NM_003161	0.6
9	CSF1	colony stimulating factor 1 (macrophage)	NM_000757	0.6
10	EGFR	epidermal growth factor receptor (erythroblastic leukemia viral (v-erb-b) oncogene homolog, avian)	NM_201283	0.6
11	MIA3	melanoma inhibitory activity family, member 3	NM_198551	0.6
12	CXCL12	chemokine (C-X-C motif) ligand 12 (stromal cell-derived factor 1)	NM_001033886	0.6
13	ARHGAP5	Rho GTPase activating protein 5	NM_001030055	0.6
14	F2RL1	coagulation factor II (thrombin) receptor-like 1	NM_005242	0.6
15	VEGFA	vascular endothelial growth factor A	NM_001025366	0.6
16	BCL2	B-cell CLL/lymphoma 2	NM_000633	0.6
17	FGF2	fibroblast growth factor 2 (basic)	NM_002006	0.6
18	INSR	insulin receptor	NM_000208	0.6
19	PTP4A1	protein tyrosine phosphatase type IVA, member 1	NM_003463	0.6
20	VEGFC	vascular endothelial growth factor C	NM_005429	0.6
21	JAK2	Janus kinase 2	NM_004972	0.5
22	LAMB1	laminin, beta 1	NM_002291	0.5
23	INS	insulin-like growth factor 2 (somatomedin A); insulin; INS-IGF2 readthrough transcript	NM_000207	0.5
24	IL6ST	interleukin 6 signal transducer (gp130, oncostatin M receptor)	NM_175767, NM_002184	0.5
25	F2R	coagulation factor II (thrombin) receptor	NM_001992	0.5
26	PDPN	podoplanin	NM_198389	0.4
27	PIK3R1	phosphoinositide-3-kinase, regulatory subunit 1 (alpha)	NM_181523	0.3

Supplementary Table 3. List of transcription regulators conserved in transcription factor binding site of migration/invasion-associated genes.

The each transcription regulators found onto S1PR1, EDN2, THBS1, and HB-EGF binding regions are listed by Encyclopedia of DNA Elements (ENCODE) dataset in the Genome Brower (http://genome.ucsc.edu/cgi-bin/hgGateway). Among the several transcription regulator of S1PR1 (the number of 19), EDN2 (the number of 11), THBS1 (the number of 20), and HB-EGF (the number of 36), Rad21 is found onto S1PR1, EDN2, THBS1, and HB-EGF binding sites.

**S1PR1** (#19)	**EDN2** (#11)	**THBS1** (#20)	**HB-EGF** (#36)
BCLAF1	AP-2gamma	c-Myc	BCL11A
c-Fos	c-Myc	CTCF	CCNT2
CTCF	CTCF	ELF1	CEBPB
EBF	ER-α	ER-α	c-Fos
EBF1	GR	FOXA1	c-Myc
Egr-1	HA-E2FQ	GR	CTCF
ELF1	Ini1	HEY1	E2F6
HDAC2	NF-kB	HNF4A	EBF
JunD	Pol2	JunD	EBF1
KAP1	Rad21	NRSF	Egfp-fos
NRSF	TBP	Pol2	Egr-1
PAX5		Rad21	FOSL1
Pol2		Sin3AK	FOSL2
Rad21		SMC3	HA-E2F1
RXRA		SP1	HDAC2
SMC3		TAF1	HEY1
TAF1		TAF7	HNF4A
TBP		TBP	HNF-4G
ZNF263		USF1	JunD
		ZEB1	Max
			MEF2A
			MEF2C
			NF-kB
			NRSF
			Pol2
			Rad21
			RPC155
			SP1
			SRF
			STAT3
			TAF1
			TAF7
			TBP
			TRL1
			ZBTB7A
			ZNF263

Supplementary Figure legends

Supplementary Figure S1. siRNA-mediated Rad21 knockdown in SKOV3^{R248} cells

The protein levels of Rad21 were determined by western blot analysis after transfection with control siRNA or Rad21 siRNA in SKOV3^{R248} cells. β-Actin was used as an internal control.

Supplementary Figure S2. siRNA-mediated S1PR1 and THBS1 knockdown in SKOV3^{R248} cells

The protein levels of S1PR1 and THBS1 were determined by western blot analysis after transfection with control, S1PR1, or THBS1 siRNA in SKOV3^{R248} cells. β-Actin was used as an internal control.

References

1 Consortium, E. P. An integrated encyclopedia of DNA elements in the human genome. *Nature* **489**, 57-74, doi:10.1038/nature11247 (2012).

2 Consortium, E. P. A user's guide to the encyclopedia of DNA elements (ENCODE). *PLoS Biol.* **9**, e1001046, doi:10.1371/journal.pbio.1001046 (2011).

3 Wang, J. *et al.* Factorbook.org: a Wiki-based database for transcription factor-binding data generated by the ENCODE consortium. *Nucleic Acids Res.* **41**, D171-176, doi:10.1093/nar/gks1221 (2013).

Supplementary Figure S1.

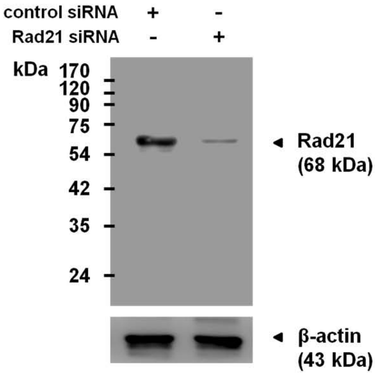

Supplementary Figure S2.

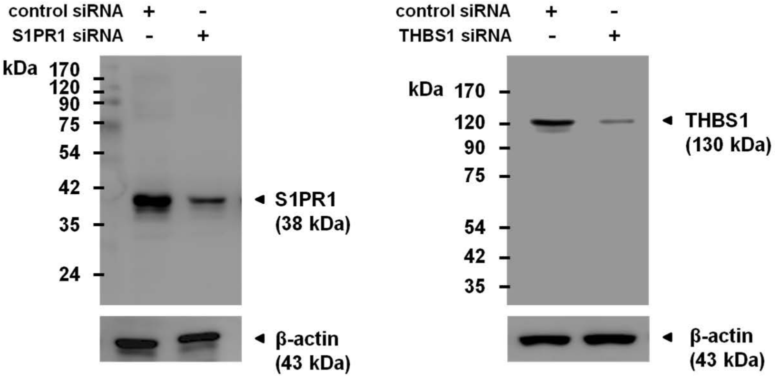

Supplement: Supplementary file 1 — Suppementary information [file 41598_2017_8880_MOESM1_ESM.pdf]
