# Supplementary material for: Mutant p53 stimulates cell invasion through an interaction with Rad21 in human ovarian cancer cells
Source: Sci Rep. 2017 Aug 22;7:9076. doi: 10.1038/s41598-017-08880-4 (PMC5567302; doi:10.1038/s41598-017-08880-4)

Figure 1

A stable transfection

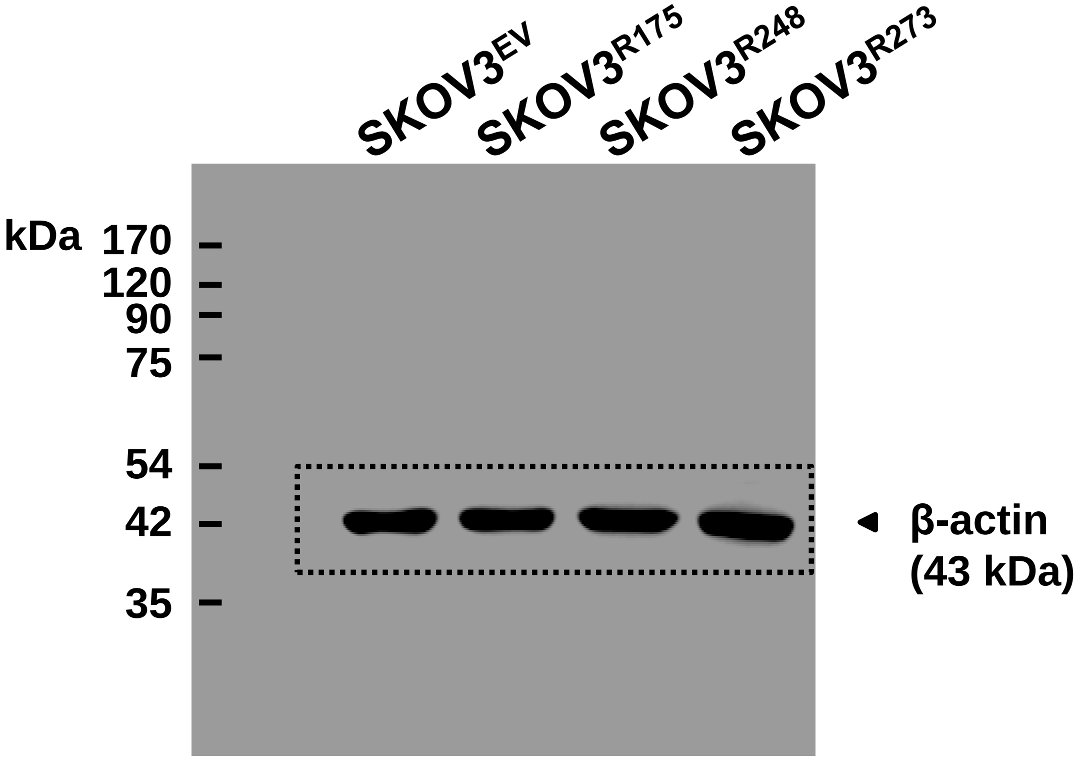

Figure 1

B   transient transfection

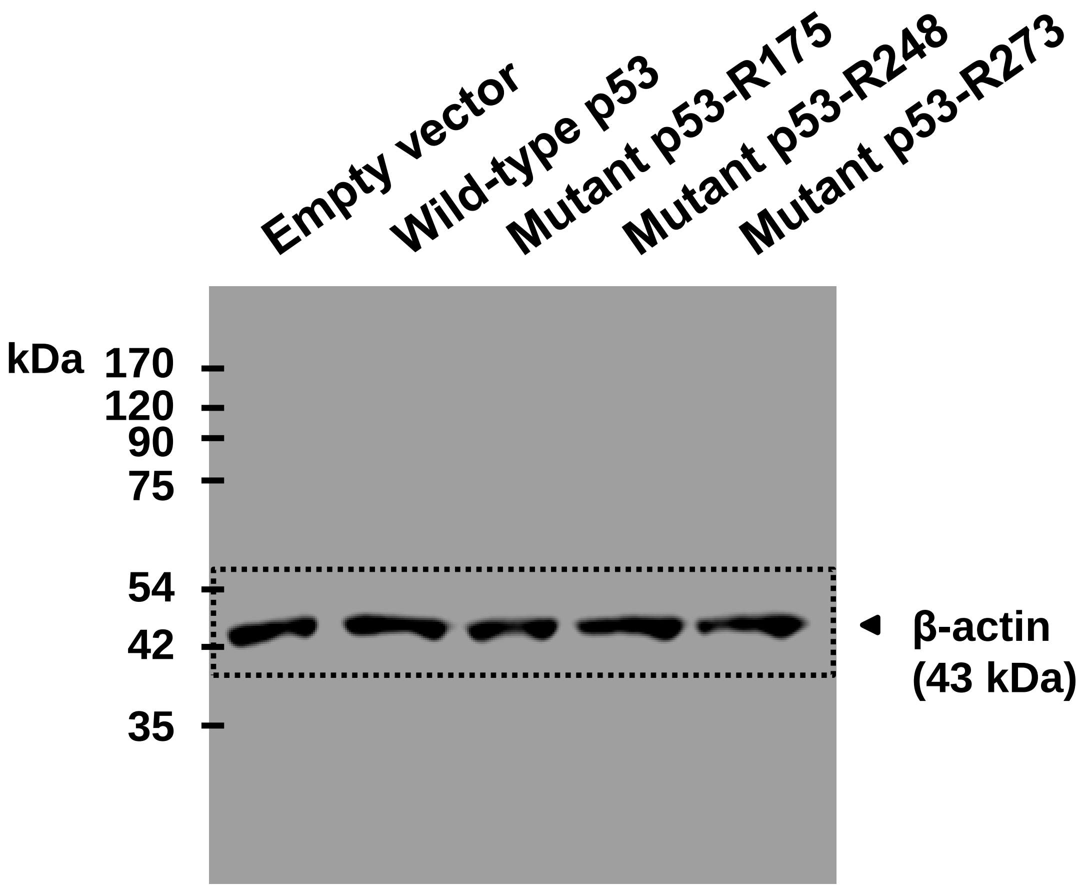

## Figure 2A

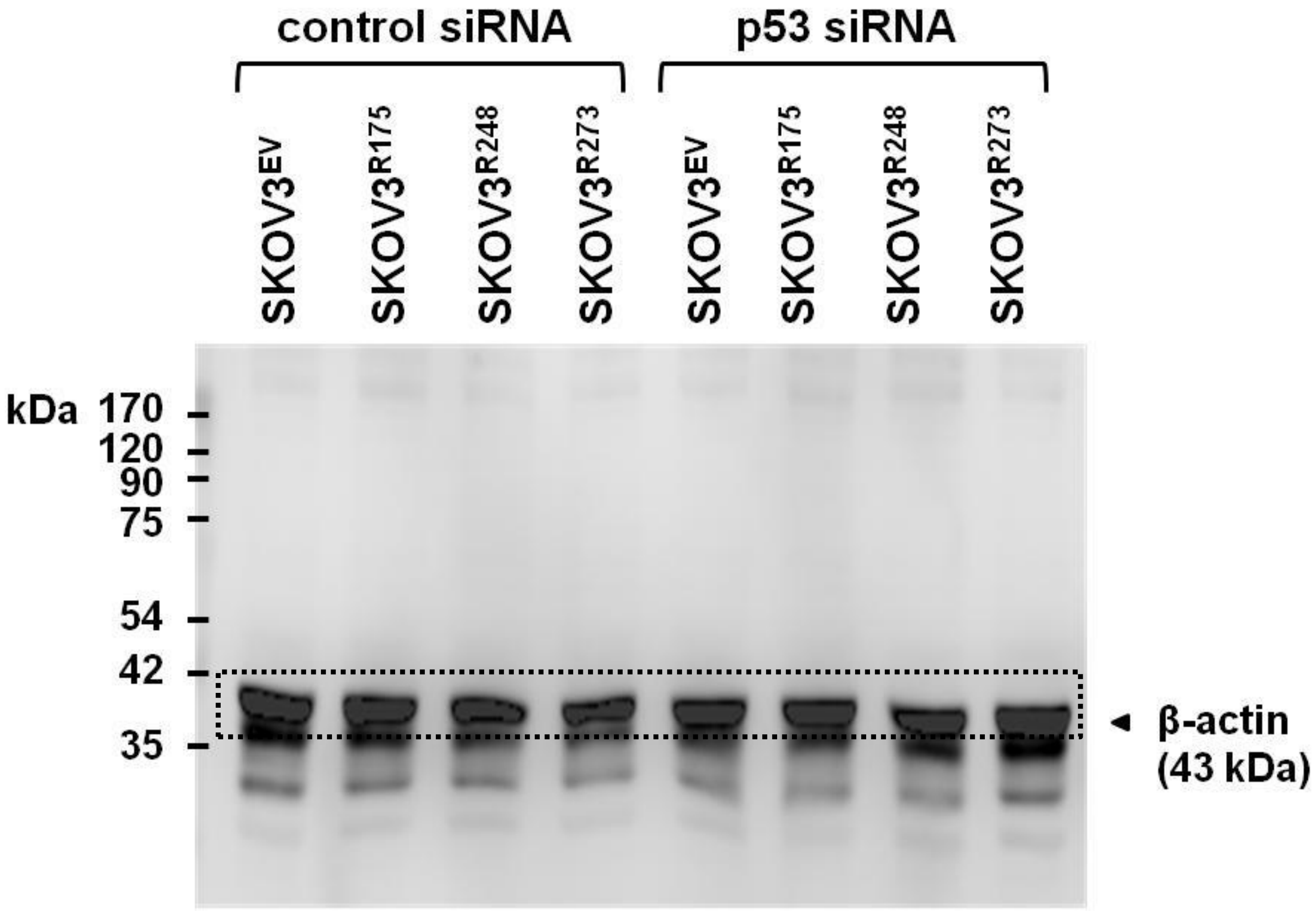
control siRNA
p53 siRNA
SKOV3EV
SKOV3R175
SKOV3R248
SKOV3R273
SKOV3EV
SKOV3R175
SKOV3R248
SKOV3R273
kDa
170
120
90
75
54
42
35
β-actin
(43 kDa)

# Figure 4B_actin for S1PR1 blot

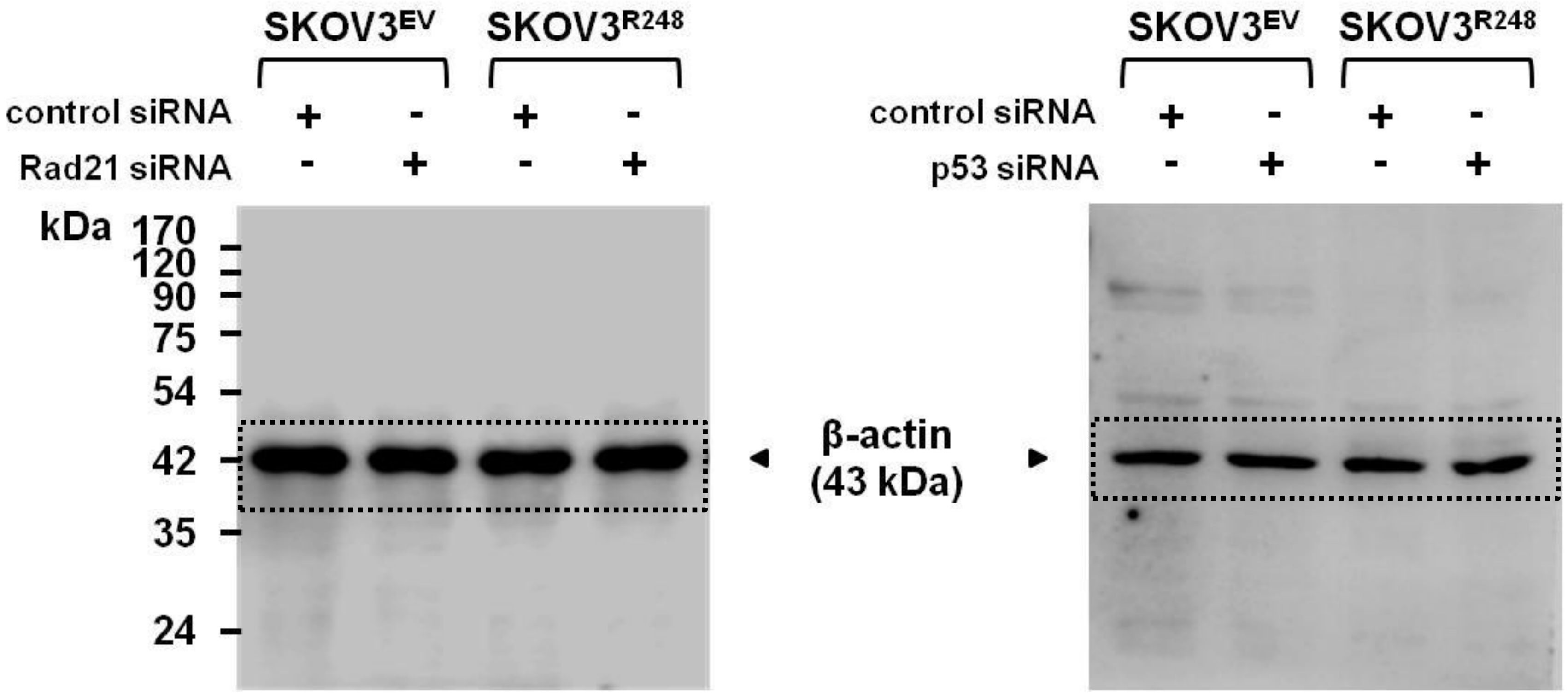

# Figure 4B_actin for THBS1 blot

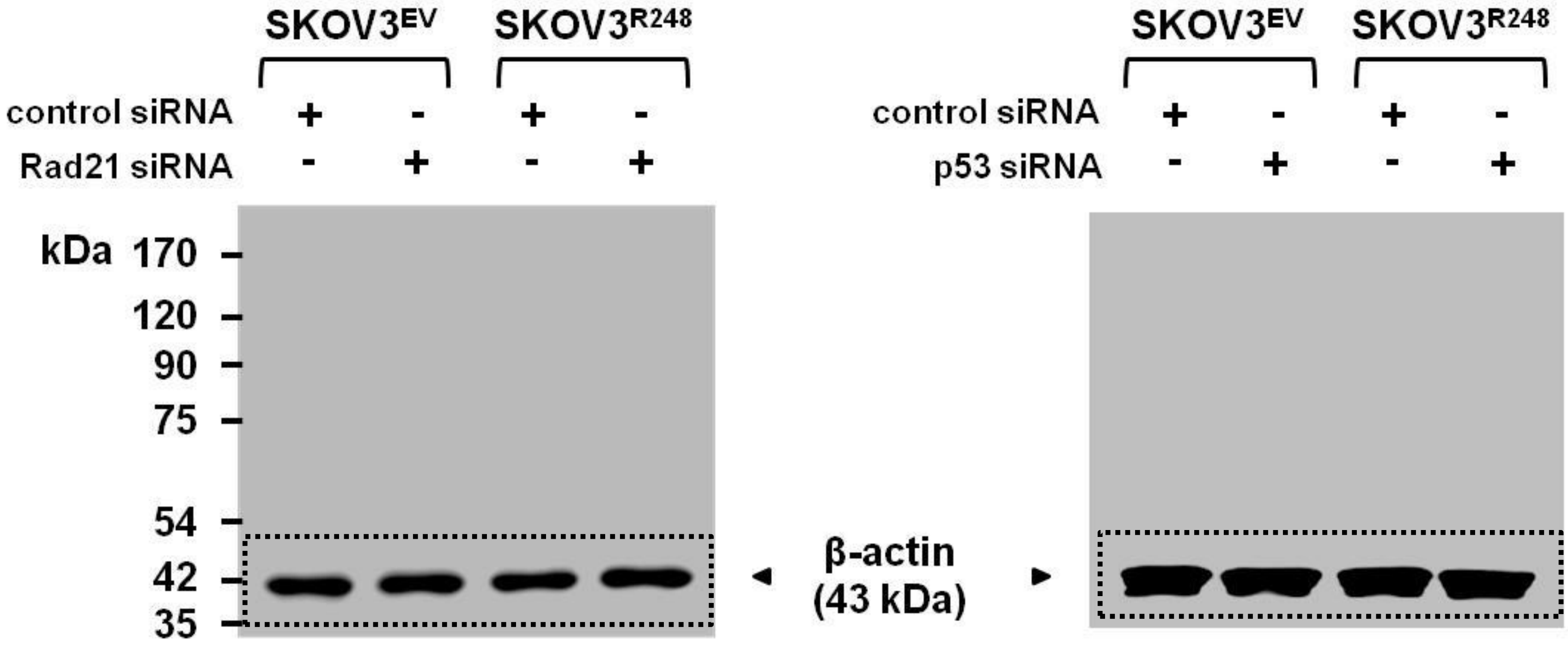

**Figure 5A_IP:p53, IB:p53**

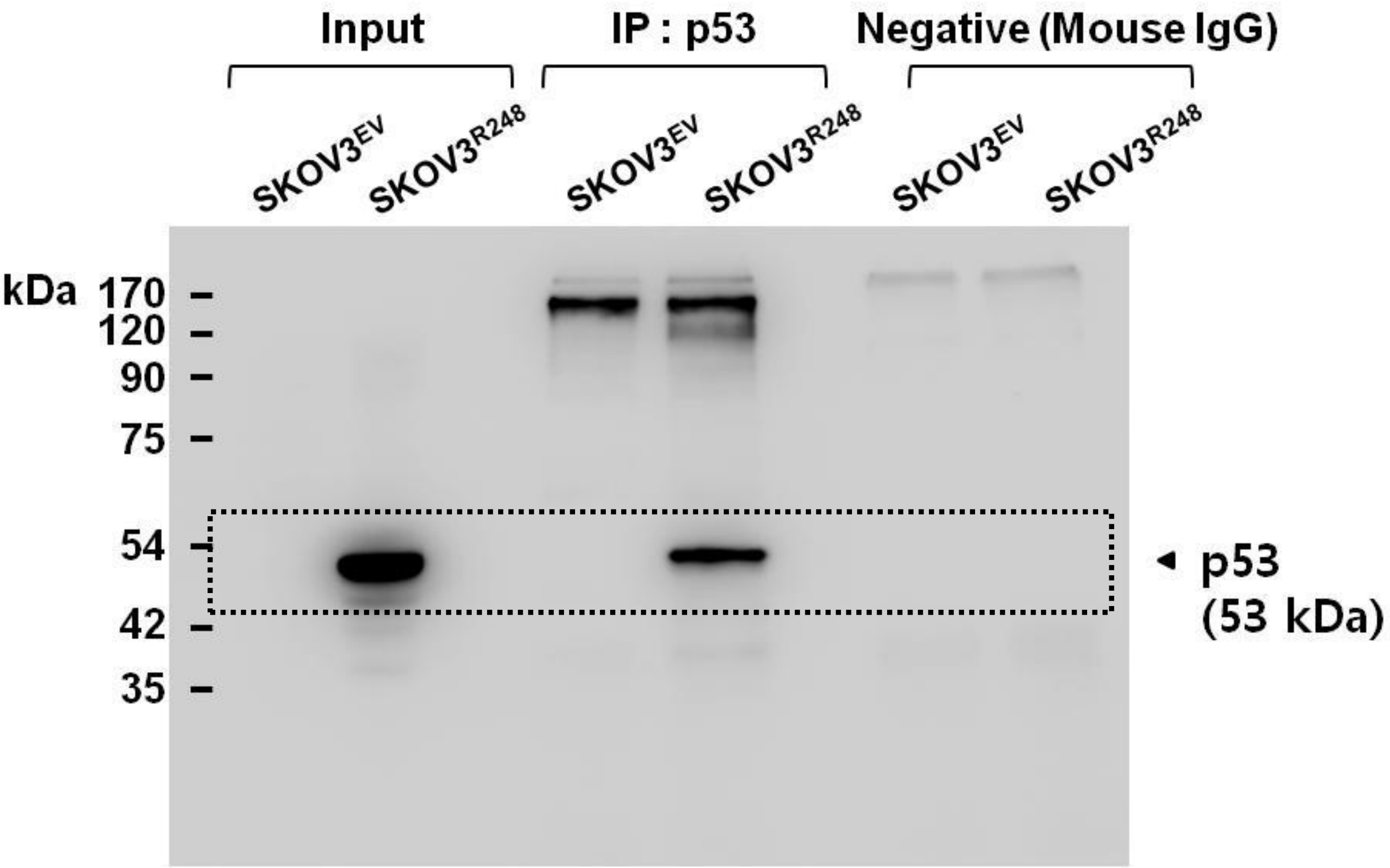

**Figure 5A_IP:p53, IB:Rad21**

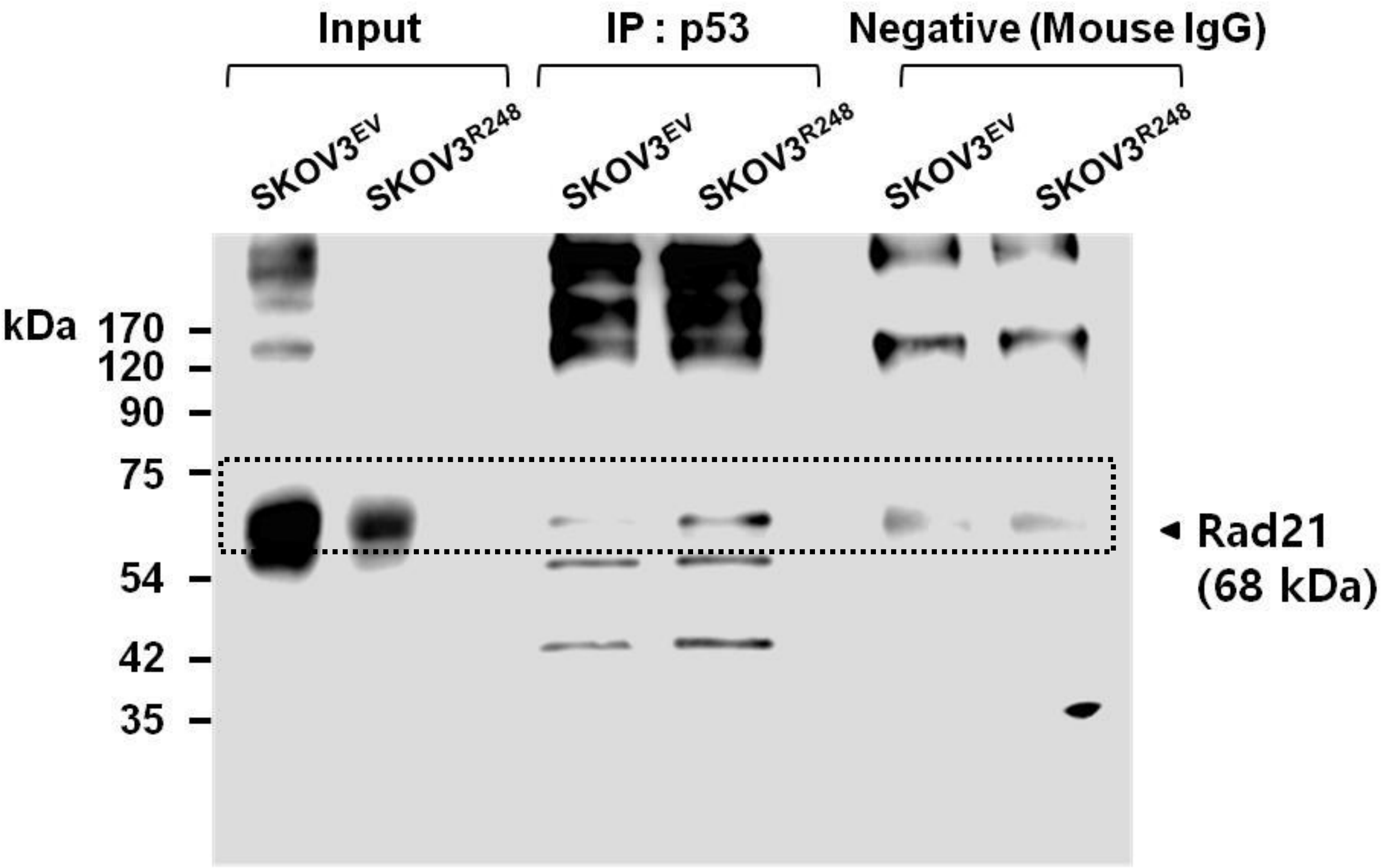

**Figure 5A_IP:Rad21, IB:Rad21**

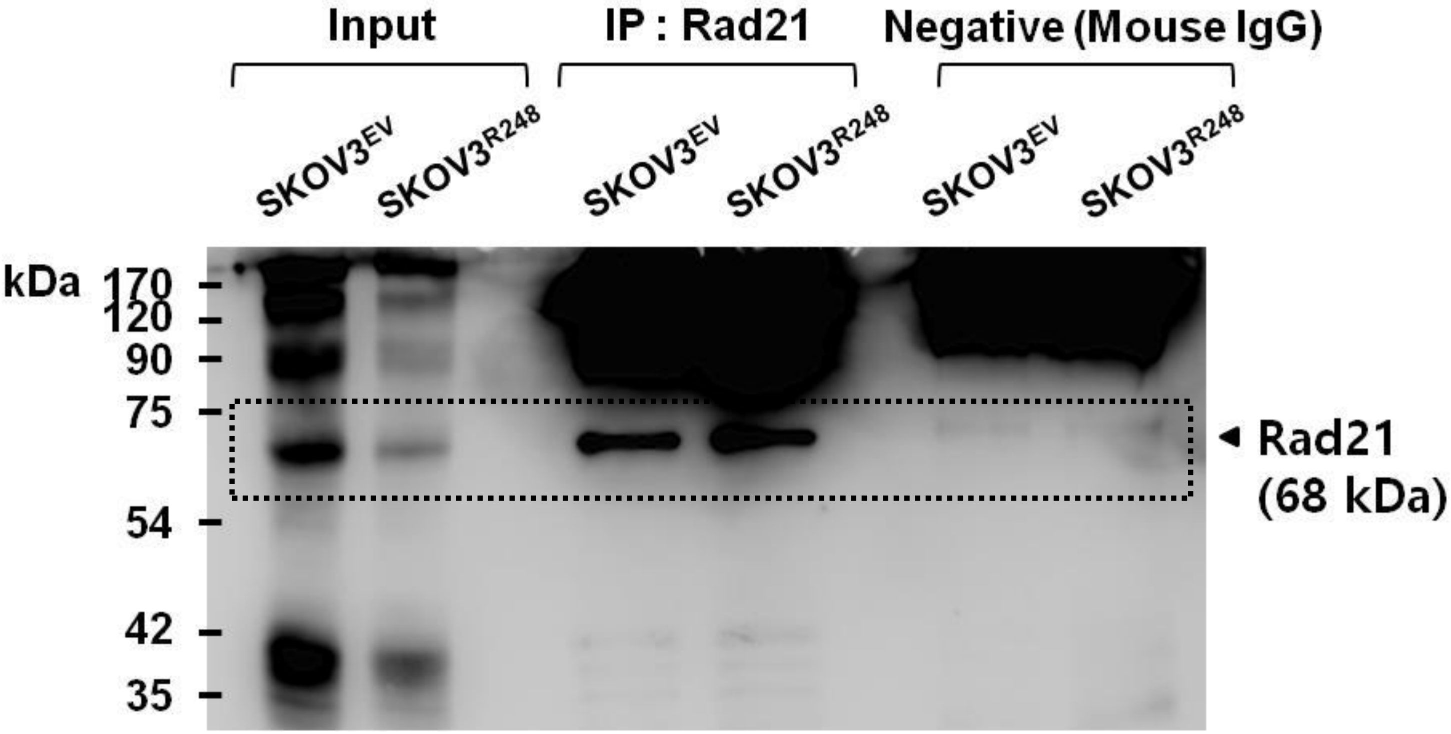

Figure 5A_IP:Rad21, IB:p53

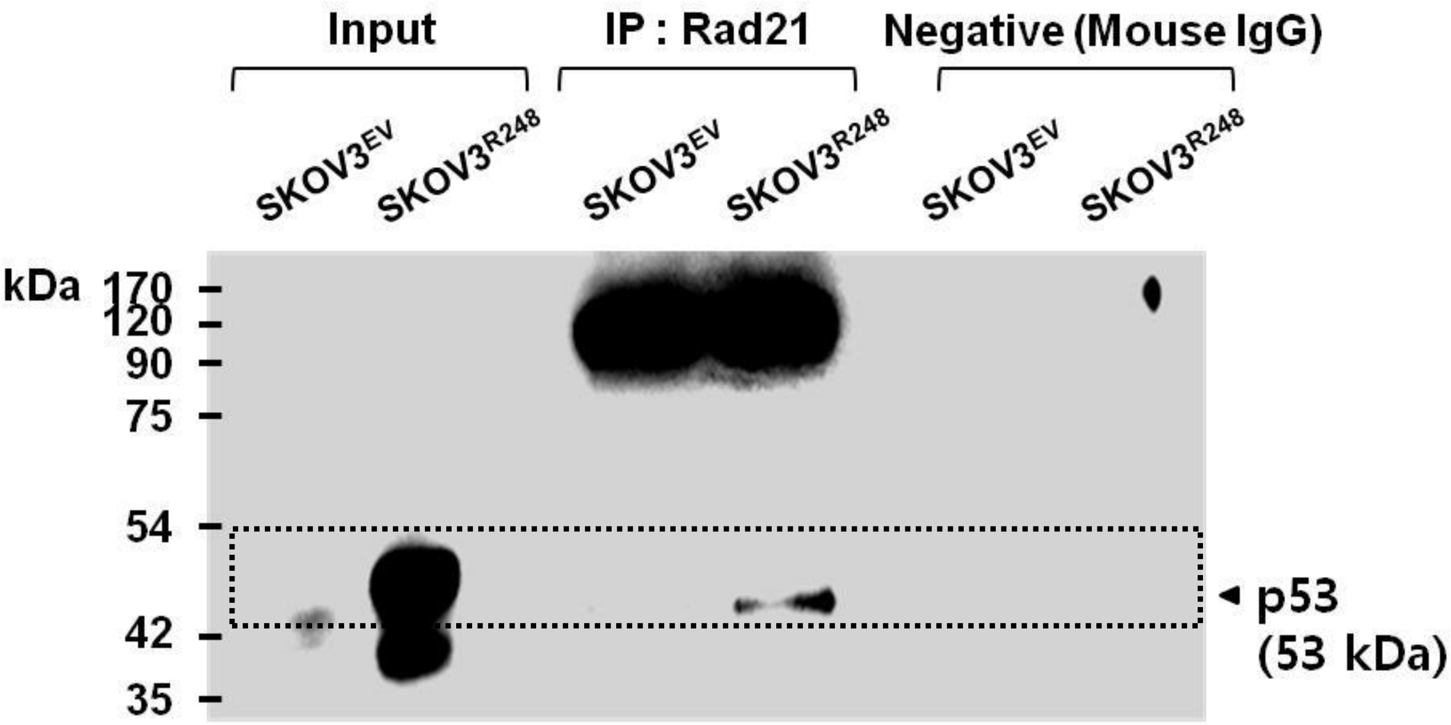

Supplement: Supplementary file 2 — Figure S1 [file 41598_2017_8880_MOESM2_ESM.pdf]
